# MMP-10/Stromelysin-2 Promotes Invasion of Head and Neck Cancer

**DOI:** 10.1371/journal.pone.0025438

**Published:** 2011-10-05

**Authors:** Elsayed Mohamed Deraz, Yasusei Kudo, Maki Yoshida, Mariko Obayashi, Takaaki Tsunematsu, Hirotaka Tani, Samadarani B. S. M. Siriwardena, Mohammad Reza Kiekhaee, Guangying Qi, Shinji Iizuka, Ikuko Ogawa, Giuseppina Campisi, Lorenzo Lo Muzio, Yoshimitsu Abiko, Akira Kikuchi, Takashi Takata

**Affiliations:** 1 Division of Frontier Medical Science, Department of Oral and Maxillofacial Pathobiology, Graduate School of Biomedical Sciences, Hiroshima University, Hiroshima, Japan; 2 Center of Oral Clinical Examination, Hiroshima University Hospital, Hiroshima, Japan; 3 Department of Stomatological Sciences, University of Palermo, Palermo, Italy; 4 Department of Surgical Sciences, University of Foggia, Foggia, Italy; 5 Department of Biochemistry, School of Dentistry at Matsudo, Nihon University, Chiyoda, Japan; 6 Department of Molecular Biology and Biochemistry, Osaka University Graduate School of Medicine, Suita, Japan; Karolinska Institute, Sweden

## Abstract

**Background:**

Periostin, IFN-induced transmembrane protein 1 (IFITM1) and Wingless-type MMTV integration site family, member 5B (Wnt-5b) were previously identified as the invasion promoted genes of head and neck squamous cell carcinoma (HNSCC) by comparing the gene expression profiles between parent and a highly invasive clone. We have previously reported that Periostin and IFITM1 promoted the invasion of HNSCC cells. Here we demonstrated that Wnt-5b overexpression promoted the invasion of HNSCC cells. Moreover, stromelysin-2 (matrix metalloproteinase-10; MMP-10) was identified as a common up-regulated gene among Periostin, IFITM1 and Wnt-5b overexpressing HNSCC cells by using microarray data sets. In this study, we investigated the roles of MMP-10 in the invasion of HNSCC.

**Methods and Findings:**

We examined the expression of MMP-10 in HNSCC cases by immunohistochemistry. High expression of MMP-10 was frequently observed and was significantly correlated with the invasiveness and metastasis in HNSCC cases. Next, we examined the roles of MMP-10 in the invasion of HNSCC cells *in vitro*. Ectopic overexpression of MMP-10 promoted the invasion of HNSCC cells, and knockdown of MMP-10 suppressed the invasion of HNSCC cells. Moreover, MMP-10 knockdown suppressed Periostin and Wnt-5b-promoted invasion. Interestingly, MMP-10 overexpression induced the decreased p38 activity and MMP-10 knockdown induced the increased p38 activity. In addition, treatment with a p38 inhibitor SB203580 in HNSCC cells inhibited the invasion.

**Conclusions:**

These results suggest that MMP-10 plays an important role in the invasion and metastasis of HNSCC, and that invasion driven by MMP-10 is partially associated with p38 MAPK inhibition. We suggest that MMP-10 can be used as a marker for prediction of metastasis in HNSCC.

## Introduction

Head and neck squamous cell carcinoma (HNSCC) is one of the most common types of human cancer, with an annual incidence of more than 500,000 cases worldwide [1]. Despite the advances of treatment strategies, the mortality rate and failure of treatment options in HNSCC patients are still high. High mortality and poor prognosis of HNSCC are predicted by occurrence of lymph node metastasis, which is a common event in cancer patients [2]. HNSCC develops through the accumulation of multiple genetic and epigenetic alterations in a multi-step process [3]. Attempts to identify the genes involved in the metastasis are pivotal for the early prediction of HNSCC behavior. The process of metastasis consists of sequential and selective steps including proliferation, induction of angiogenesis, detachment, motility, invasion into circulation, aggregation and survival in the circulation, cell arrest in distant capillary beds and extravasation into organ parenchyma [4], [5]. The identification of novel invasion and metastasis related molecules in HNSCC and the better understanding of the mechanisms by which carcinoma cells undergo during the steps of invasion and metastasis are of fundamental importance to design better therapeutic strategies for treating this disease.

Gene expression microarrays have been emerged and their widespread use has led to the identification of potential candidate genes. Further investigation of the biological behaviors and significant clinical outcome of these genes particularly in HNSCC is of great interest. We previously established a HNSCC cell line, MSCC-1, from lymph node metastasis [6]. Moreover, we isolated a highly invasive clone MSCC-Inv1 from MSCC-1 cells by using an *in vitro* invasion assay device [7]. Then, we compared the transcriptional profile of parent cells (MSCC-1) and a highly invasive clone (MSCC-Inv1) by microarray analysis in order to identify genes that differ in their expression [8]. Several genes were selectively overexpressed in the highly invasive clone. Among these genes, Periostin (osteoblast-specific factor 2 (fasciclin I like)) was the most highly expressed gene and the second was IFITM1 (IFN-induced transmembrane protein 1). In fact, we demonstrated that Periostin and IFITM1 promoted invasion both *in vitro* and *in vivo* [8], [9]. We also identified Wingless-type MMTV integration site family, member 5B (Wnt-5b) as the third highly expressed gene in MSCC-Inv1. Here, we confirmed the capability of Wnt-5b to promote the invasion of HNSCC cells *in vitro.* Moreover, we identified matrix metalloproteinase-10 (MMP-10) as a common upregulated gene by invasion promoting molecules including Periostin, IFITM1 and Wnt-5b. Matrix metalloproteinases (MMPs) represent a family of zinc-dependent proteinases which are able to degrade ECM components such as collagens and proteoglycans and they have a role in normal development and tissue damage in various pathophysiological conditions involving arthritis, wound healing and tumor development [10]. MMPs can be classified into subgroups including; collagenases, stromelysins, gelatinases, and membrane type MMPs [11]. Some members of MMPs are implicated in the invasion and metastasis in HNSCC such as MMP-2, membrane type-1 MMP (MT1-MMP), and MMP-9 [12], [13]. Overexpression of these MMPs has been correlated with the invasion, metastasis, and poor prognosis. In the present study, we investigated the roles of MMP-10 in the invasion of HNSCC.

## Results

### Wnt-5b promotes the invasion of HNSCC

**Figure 1:**
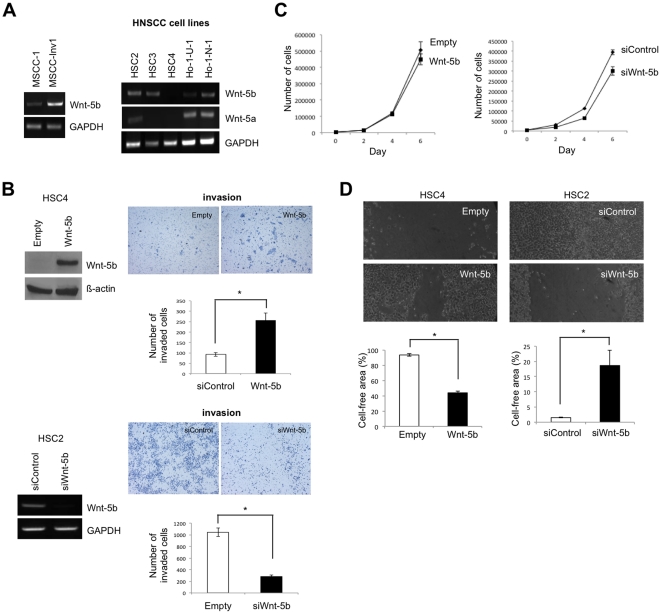
Wnt-5b promotes the invasion of HNSCC. *A,* Expression of Wnt-5b in the highly invasive clone and HNSCC cell lines. Expression of Wnt-5b mRNA in MSCC-1, MSCC-Inv1 as well as six HNSCC cell lines; HSC2, HSC3, HSC4, Ca-9-22, Ho-1-N-1, and Ho-1-U-1 was examined by RT-PCR. Expression of Wnt-5a mRNA was also examined in 6 HNSCC cell lines. GAPDH was used as a loading control. *B,* Wnt-5b promoted the invasion of HNSCC cells. For generation of Wnt-5b-overexpressing cells, Wnt-5b expression vector was transfected into HSC4 cells without Wnt-5b expression. We obtained the stable clone and the expression of Wnt-5b was examined by Western blotting with anti-Wnt-5b polyclonal antibody (left upper panel). We used empty vector transfected cells (empty) as a control. β-actin expression was used as a loading control. The invasiveness of Wnt-5b-overexpressing cells (right upper panel) was examined by *in vitro* invasion assay. 1.5×10^5^ cells were placed on the upper compartment of the cell culture insert. After 12h of incubation, the penetrated cells onto the lower side of the membrane were fixed in formalin and stained with hematoxylin. HSC2 cells with Wnt-5b expression was transfected by Wnt-5b siRNA (siWnt-5b) or control siRNA (siControl). A scrambled sequence that does not show significant homology to rat, mouse or human gene sequences was used as a control. The effect of knockdown was evaluated by RT-PCR (left lower panel). GAPDH was used as a loading control. The invasiveness of Wnt-5b-knocked-down cells was examined by *in vitro* invasion assay (right lower panel). After 24 h of incubation, the penetrated cells onto the lower side of the membrane were fixed in formalin and stained with hematoxylin. The bars show the average values and SDs of three independent experiments. *Significantly different from empty vector transfected cells or control siRNA transfected cells at *P*<0.01. *C,* Cell proliferation of Wnt-5b-overexpressing cells and Wnt-5b-knocked-down cells. Cells were plated on 24 well plates, and trypsinized cells were counted by cell counter at 0, 2, 4 and 6 day. The bars show the average values and SDs of three independent experiments. *D,* Migration of Wnt-5b-overexpressing cells and Wnt-5b-knocked-down cells. Migration of the cells was determined by wound healing assay. At 24 h after scratching the cells, phase-contrast images (10× field) of the wound healing process were photographed digitally with an inverted microscope. The distance of the wound areas were measured on the images, set at 100% for 0 h, and the mean percentage of the total distances of the wound areas was calculated. The bars show the average values and SDs of three independent experiments. *Significantly different from empty vector transfected cells or control siRNA transfected cells at *P*<0.01.

Wnt-5b is a member of the Wnt gene family, a group of secreted glycoproteins that plays an important role in oncogenesis and in several developmental processes and triggers intracellular responses through various signaling pathways. By comparing the transcriptional profile of the parent cells and the highly invasive clone by microarray analysis, Wnt-5b was the third highly expressed gene in the highly invasive clone after Periostin and IFITM1. We first examined whether Wnt-5b was involved in the invasion of HNSCC. The higher expression of Wnt-5b in the highly invasive clone compared to the parent cells was verified by RT-PCR (Figure 1A). We examined the expression of Wnt-5b mRNA in six HNSCC cell lines. Wnt-5b mRNA expression was noted in almost all of the HNSCC cell lines except HSC4 cell (Figure 1A). To clarify the role of Wnt-5b in the invasiveness of HNSCC, we generated the Wnt-5b-overexpressing cells by transfection of Wnt-5b into HSC4 cells without Wnt-5b expression. After getting the stable clone of Wnt-5b-overexpressing cells (Figure 1B), they were used for checking the invasiveness by *in vitro* invasion assay. Wnt-5b overexpression significantly enhanced the invasion of HNSCC cells *in vitro* (Figure 1B). To confirm the Wnt-5b-promoted invasion of HNSCC cells, we examined the knockdown of Wnt-5b by using siRNA in HSC2 cells with high expression of Wnt-5b. Treatment of Wnt-5b siRNA reduced the expression of Wnt-5b mRNA and significantly inhibited the invasion (Figure 1B). Although Wnt-5b did not affect cell growth (Figure 1C), it significantly promoted cell motility of HNSCC cells as demonstrated by wound healing assay (Figure 1D). Interestingly, Wnt-5b siRNA significantly inhibited cell motility of HNSCC cells (Figure 1D). Moreover, we compared the gene expression profile between control and Wnt-5b-overexpressing HSC4 cells by microarray analysis (Data S1). S100A8, SERPINB4, osteopontin and SERPINB3 were upregulated and TGF-ß2, CDH11 and thrombospondin 1 were downregulated in Wnt-5b-overexpressing cells (Table S1).

### MMP-10 is identified as a common target gene for Periostin, IFITM1 and Wnt-5b overexpression

**Figure 2:**
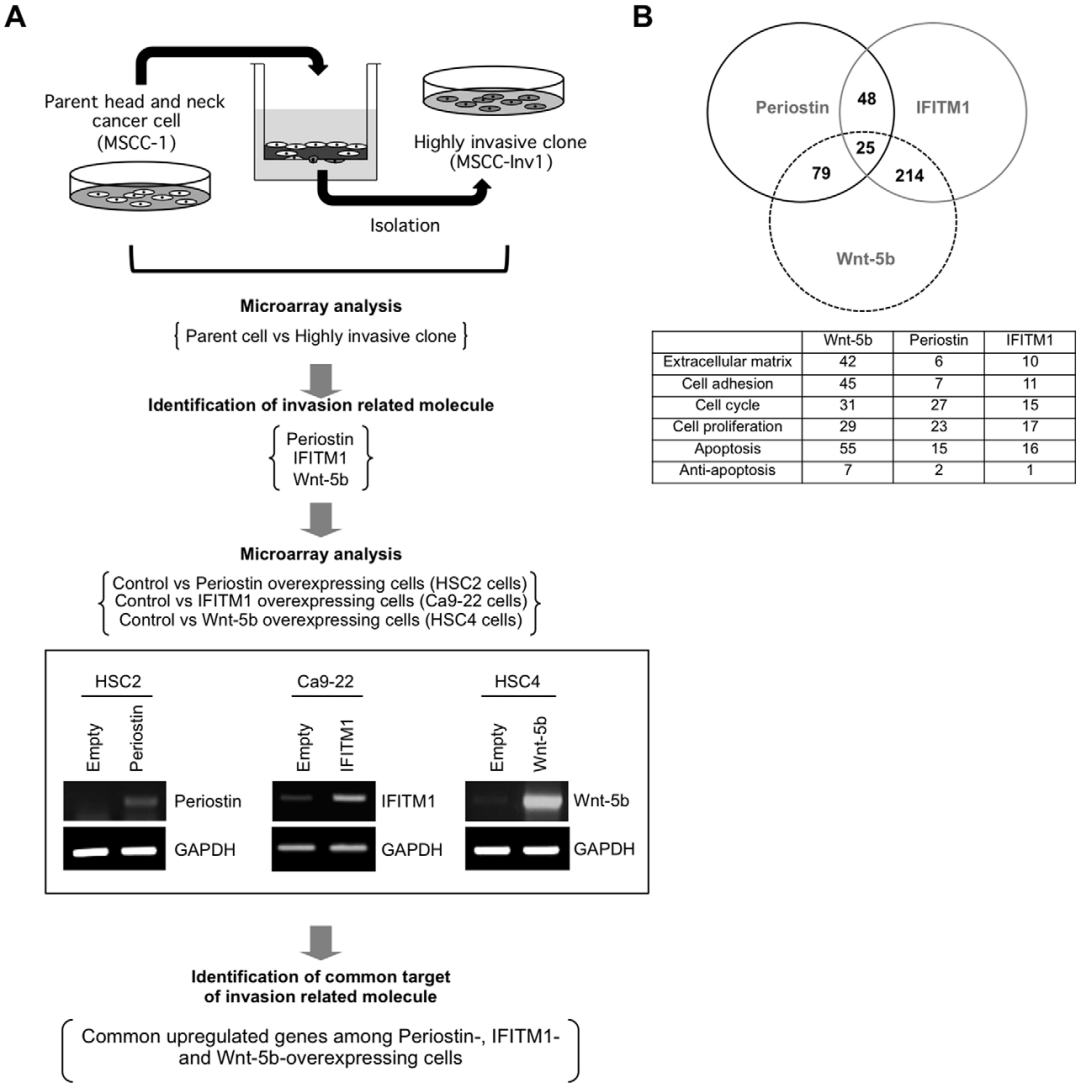
MMP-10 and MMP13 are common upregulated genes by Periostin, IFITM1, and Wnt-5b overexpression. *A,* Schema shows the strategy to identify the common target of invasion related molecules. Periostin, IFITM1, and Wnt-5b are identified as the invasion related molecules by comparing the gene expression profile between the parent (MSCC-1 cells) and a highly invasive clone (MSCC-Inv1 cells). To identify common targets of invasion related molecules, we compared the gene expression profiles of control vs. Periostin-overexpressing HSC2 cells, control vs. IFITM1-overexpressing Ca9-22 cells, and control vs. Wnt-5b-overexpressing HSC4 cells. Ectopic expression level of Periostin, IFITM1 and Wnt-5b in each cells are shown by RT-PCR. GAPDH expression was used as a loading control. *B,* Several upregulated genes were found between Periostin and IFITM1, Periostin and Wnt-5b, and Wnt-5b and IFITM1. By using Gene Ontology software, we identified the biological functions of these genes. The table shows these common upregulated genes comprising diverse families with variable biological functions.

To identify the common target genes for Periostin, IFITM1 and Wnt-5b overexpression, we compared the gene expression profiles between control HSC2 cells and Periostin-overexpressing HSC2 cells, control Ca9-22 cells and IFITM1-overexpressing Ca9-22 cells, and control HSC4 cells and Wnt-5b-overexpressing HSC4 cells (Figure 2A). As a result, several groups of genes with variable biological functions in normal development and in the process of malignancy were found to be common in Periostin, IFITM1, and Wnt-5b-overexpressing cells (Figure 2B). A number of common target genes with variable biological behaviors including cell adhesion, cell proliferation, apoptosis, cell cycle and others were identified. Gene ontology analysis was done by using Gene Spring GX software to identify the biological functions of these molecules (Figure 2B). Common up-regulated genes between Periostin- and IFITM1-overexpression, Periostin- and Wnt-5b-overexpression and IFITM1- and Wnt-5b-overexpression are listed in Table S2, S3 and S4, respectively. Several genes were the highly upregulated among Periostin-, IFITM1-, and Wnt-5b-overexpressing HNSCC cells (Table S5). Among them, stromelysin-2 (MMP-10) was included. MMPs are known as a family of zinc-dependent proteinases which are able to degrade ECM components and they have a role in tumor development [10]. However, little is known about involvement of MMP-10 in the invasion and metastasis of cancer cells. Therefore, we focused on MMP-10 as a common upregulated molecule induced by invasion related factors of HNSCC and examined its role in the invasion of HNSCC.

### High expression of MMP-10 is well correlated with invasion patterns and metastasis in HNSCC

**Figure 3:**
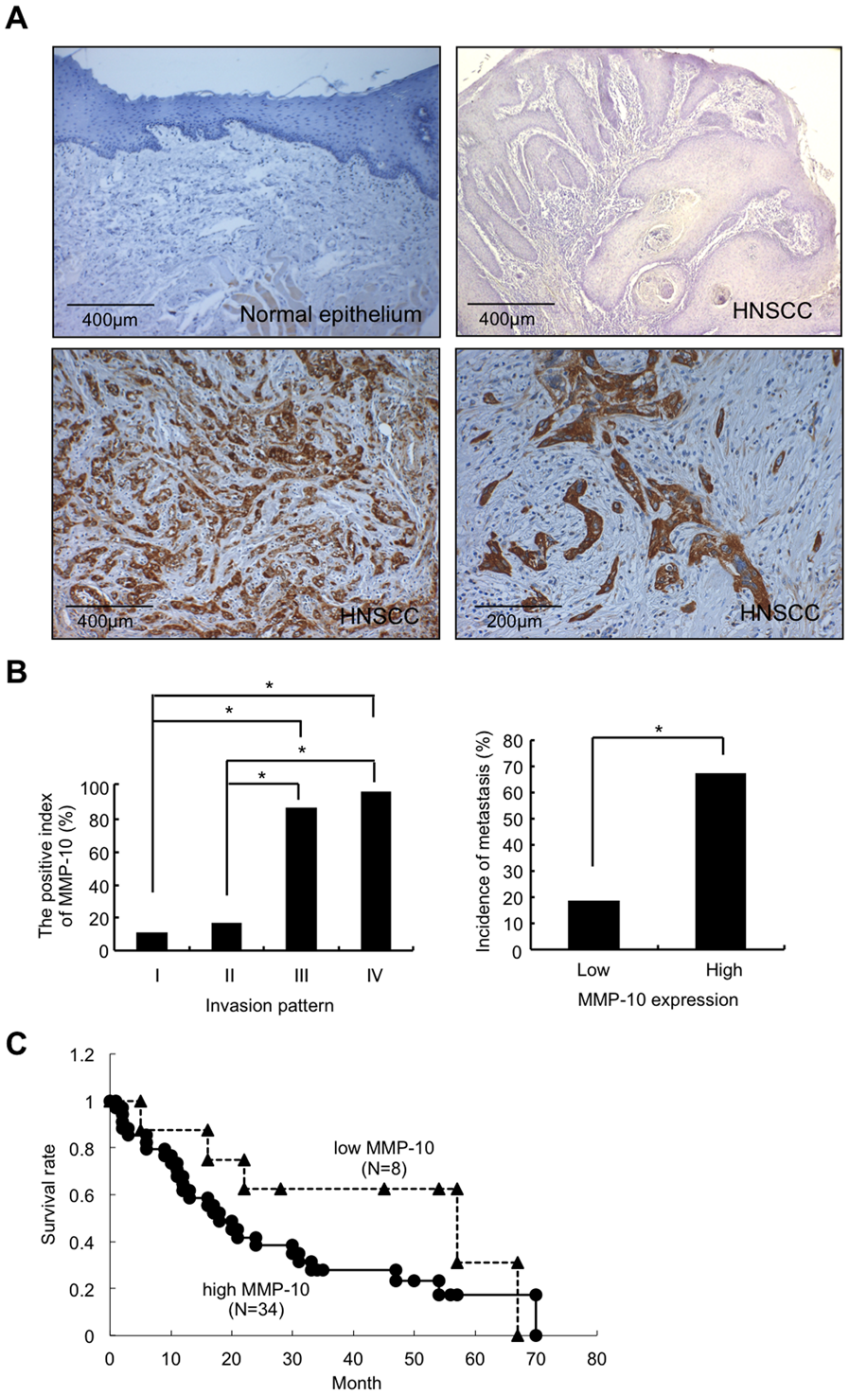
High expression of MMP-10 is observed in HNSCC cases. *A,* Immunohistochemical expression of MMP-10 in 116 HNSCC cases and 30 normal epithelia. Normal epithelium is completely negative for MMP-10 compared to HNSCC cases in which most tumor cells showed highly expression of MMP-10. Representative case of low MMP-10 expression in normal oral epithelium and HNSCC case (Jacobsson's classification Pattern I), and representative cases of high expression of MMP-10 (low and high magnification) in HNSCC cases (Jacobsson's classification Pattern IV) are shown. Scale bar is shown in each picture. *B,* Correlation between MMP-10 expression and invasion and metastasis in HNSCC cases. Left graph shows the relationship between MMP-10 expression and pattern of invasion. The Jacobsson's classification (Patterns I-IV) was used for evaluation of invasion pattern (Figure S1) [14]. *Significantly different from pattern I or II at *P*<0.01. Right graph exhibits the relationship between MMP-10 expression and metastasis. *Significantly different from low expression of MMP-10 at *P*<0.01. *C,* MMP-10 expression and poor outcome. Forty-two Italian HNSCC cases were available for survival analysis. Kaplan-Meier curves show survival of HNSCC patients with high expression of MMP-10 (•, N = 34) or low expression of MMP-10 (▴, N = 8).

We first examined the expression of MMP-10 in 30 normal oral mucosal tissues and 116 HNSCC cases by immunohistochemistry. Clinical information of 116 patients including age, location, tumor size, invasion pattern, metastasis, tumor stage, TNM classification, treatment, survival and MMP-10 expression is shown in Data S2. To demonstrate the antibody specificity of immunohistochemical staining of MMP-10, we performed immunohistochemical staining without secondary antibody or without primary antibody as a negative control (Figure S1). High expression of MMP-10 was observed in 89 of 116 (76.7%) HNSCC cases, whereas non-neoplastic epithelium did not show MMP-10 expression (Figure 3A). Then, we compared the MMP-10 expression with invasion pattern, stage grouping and lymph node metastasis (Table 1). We used the Jacobsson's classification (Patterns I-IV) for evaluation of invasion pattern (Figure S2) [14]. Out of 116 HNSCC cases, 9, 12, 62, and 33 cases showed the pattern I, II, III, and IV, respectively (Table 1). Interestingly, the incidence of MMP-10 positive cases in the pattern III and IV was significantly higher than that in the pattern I and II (Figure 3B). It is well known that patterns III and IV are corresponding to poor differentiation and high metastatic rate [14]. The correlation between MMP-10 expression and invasion pattern was statistically significant (P<0.001) (Table 1). We also compared MMP-10 expression with group staging. Forty-nine HNSCC cases were available for stage grouping (I-IV) according to the criteria of the Japan Society for Head and Neck Cancer [15]. Most cases (97.1 %) showed high expression of MMP-10 in advanced stage group (III and IV), while 46.7 % of cases showed high expression of MMP-10 in early stage group (I and II). The correlation between MMP-10 expression and stage grouping was statistically significant (P<0.001) (Table 1). In addition, MMP-10 expression was significantly correlated with lymph node metastasis (P<0.001) (Table 1 and Figure 3B). Twenty-nine of 89 HNSCC cases with high expression of MMP-10 had lymph node metastasis, while 5 of 27 HNSCC cases with low expression of MMP-10 had lymph node metastasis (Figure 3B). Moreover, we examined the correlation between MMP-10 expression and survival in HNSCC cases. Forty-two HNSCC cases were available for survival analysis. Interestingly, although there was no statistical significance (*P* = 0.21), patients with high expression of MMP-10 tended to show poor prognosis (Figure 3C).

**Table 1:** Correlation between MMP-10 expression and clinico-pathologic findings in HNSCC.

		MMP-10 expression		
	No. of cases	Low	High	*P* value
**Non-neoplastic epithelium**	30	28(93.3%)	2(6.7%)	P<0.001
**HNSCC**	116	27(23.3%)	89(76.7%)	
*Pattern of invasion*				
Grade I	9	8(88.9%)	1(11.1%)	P<0.001
Grade II	12	10(83.3%)	2(16.7%)	
Grade III	62	8(12.9%)	54(87.1%)	
Grade IV	33	1(3%)	32(97.0%)	
*Stage grouping*				
I & II	15	8(53.5%)	7(46.7%)	P<0.001
III & IV	34	1(2.9%)	33(97.1%)	
*Metastasis*				
−	51	22(43.1%)	29(56.9%)	P<0.001
+	65	5(7.7%)	60(92.3%)	

### MMP-10 promotes invasion of HNSCC

**Figure 4:**
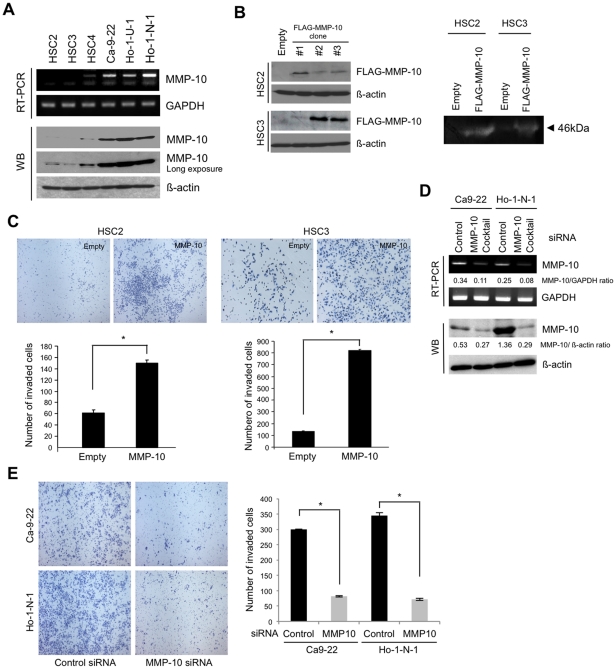
MMP-10 enhances the invasion of HNSCC cells. *A,* Expression of MMP-10 mRNA and protein in six HNSCC cell lines. Expression of MMP-10 mRNA in HSC2, HSC3, HSC4, Ca-9-22, Ho-1-N-1, and Ho-1-U-1 was examined by RT-PCR. GAPDH was used as a loading control. MMP-10 protein expression level was evaluated in the six HNSCC cell lines by Western blot analysis. Images of short and long exposure of MMP-10 protein expression are shown. ß-actin was used as a loading control. *B,* Generation of MMP-10-overexpressing cells. We obtained several clones by transfection with pBICEP-FLAG-tagged MMP-10 in HSC2 and HSC3 cells. Ectopic expression of MMP-10 was determined by Western blotting with anti-FLAG antibody (left panel). β-actin was used as a loading control. Enzymatic activity of MMP-10 was detected by stromelysin zymography (right panel). Active form of MMP-10 (arrow head) was detected in conditioned media of MMP-10-overexpressing cells. *C,* Invasive activity of MMP-10-overexpressing HSC2 (left panel) and HSC3 (right panel) cells in comparison with empty vector transfected HSC2 and HSC3 cells (empty) by *in vitro* invasion assay. Cells were fixed after incubation of 12 h or 20 h in HSC2 cells or HSC3 cells, respectively. The figure shows the stained lower side of the membrane where the cells penetrated (upper panel). The graphs show the number of invaded cells in MMP-10-overexpressing and empty vector transfected cells (lower panel). The bars show the average values and SDs of three independent experiments. *Significantly different from empty vector transfected cells at *P*<0.01. *D,* MMP-10 siRNA suppressed the invasion of HNSCC cells. Cocktail of 3 different MMP-10 siRNAs was transiently transfected into Ca-9-22 and Ho-1-N-1 cells with MMP-10 expression. A scrambled sequence that does not show significant homology to rat, mouse or human gene sequences was used as a control. After 48 h of transfection, expression of MMP-10 mRNA and protein was examined by RT-PCR and Western blotting (WB), respectively. GAPDH mRNA and β-actin protein were used as a loading control. Densitometric analysis of MMP-10 expression was performed. MMP-10/GAPDH ratio is shown. *E,* Suppression of invasion by MMP-10 knockdown in Ca9-22 and Ho-1-N-1 cells. The invasiveness of MMP-10 knocked-down cells was examined by *in vitro* invasion assay in comparison with control siRNA transfected cells. A scrambled sequence that does not show significant homology to rat, mouse or human gene sequences was used as a control. After 24 h incubation, cells were fixed and the number of invaded cells was counted. The figure shows the stained lower side of the membrane where the cells penetrated (left panel). The graph shows the number of invaded cells (right panel). The bars show the average values and SDs of three independent experiments. *Significantly different from control siRNA transfected cells at *P*<0.01.

We next examined MMP-10 mRNA and protein in 6 HNSCC cell lines by RT-PCR and Western blot analysis, respectively. High expression of MMP-10 mRNA and protein was observed in Ca9-22, Ho-1-U-1 and Ho-1-N-1 cells (Figure 4A). In HSC2, HSC3 and HSC4 cells, MMP-10 expression was low (Figure 4A). MMP-10 protein expression corresponded to mRNA expression level. To investigate whether MMP-10 promotes the invasion of HNSCC *in vitro*, we generated MMP-10-overexpressing cells by using HSC2 and HSC3 cells with low expression of MMP-10 (Figure 4B). We confirmed the higher activity of MMP-10 in conditioned media of MMP-10-overexpressing cells by stromelysin zymography (Figure 4B). Although MMP-10 overexpression did not affect the cell proliferation (data not shown), it dramatically enhanced the invasive activity in both HSC2 and HSC3 cells (*P*<0.05) (Figure 4C). To further confirm the MMP-10-mediated invasion of HNSCC cells, we examined the knockdown of MMP-10 by using siRNA in Ca-9-22 and Ho-1-N-1 cells with high expression of MMP-10. For MMP-10 knockdown, we used 3 different siRNAs (#1, #2 and #3). All siRNAs including cocktail of 3 siRNAs reduced MMP-10 expression (Figure S3). We used cocktail of 3 siRNAs in the following studies. MMP-10 knockdown inhibited the expression of MMP-10 mRNA and protein in Ca9-22 and Ho-1-N-1 cells (Figure 4D). MMP-10 knockdown significantly suppressed the invasion of HNSCC cells (Figure 4E). Taken all together, these findings indicate that MMP-10 plays an important role in the invasion of HNSCC cells.

### MMP10 knockdown suppresses the Periostin and Wnt-5b-promoted invasion of HNSCC cells

**Figure 5:**
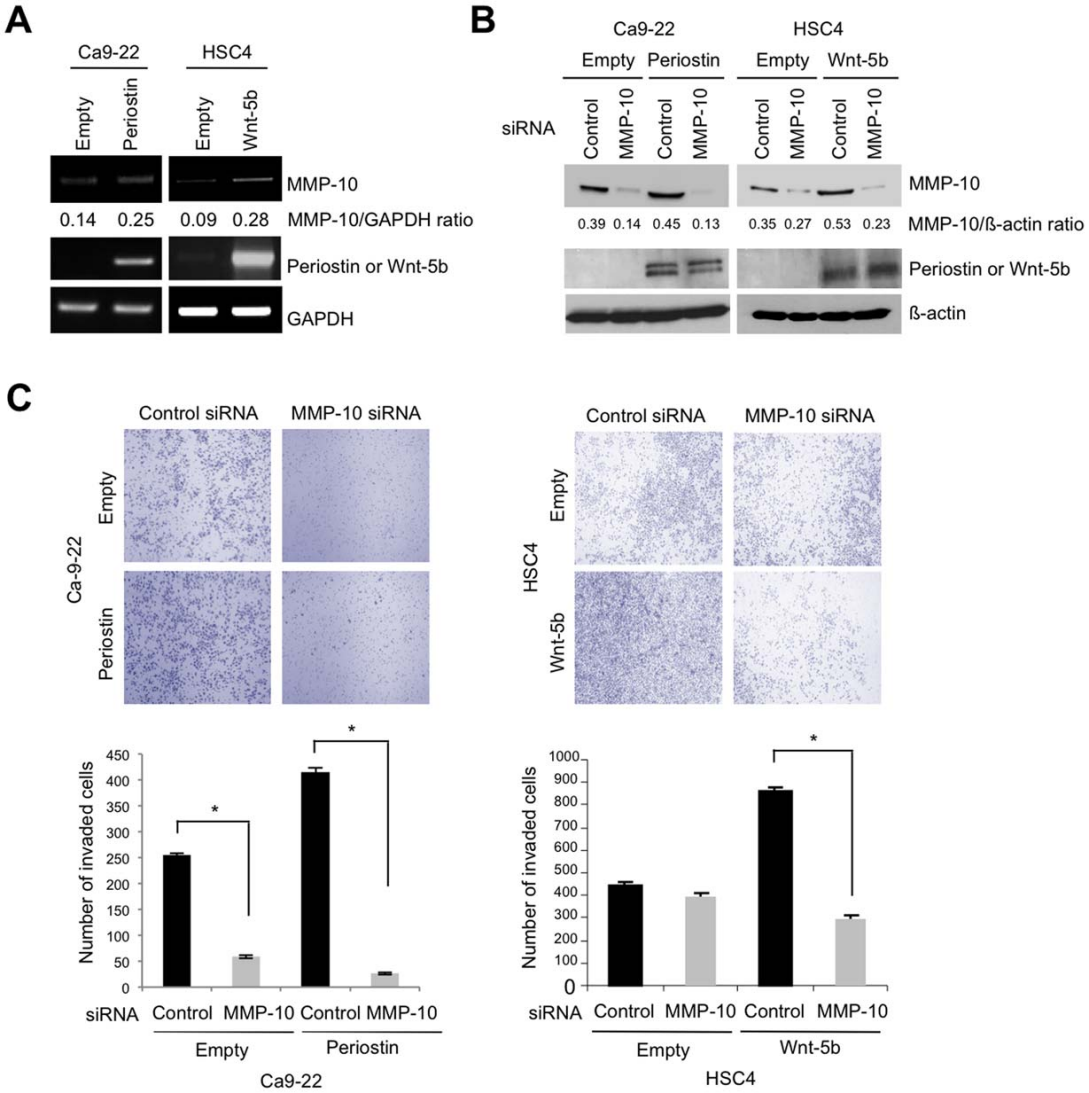
MMP-10 knockdown inhibits Periostin and Wnt-5b-promoted invasion. *A,* The expression of MMP10 mRNA was examined by RT-PCR in control Ca9-22 cells, Periostin-overexpressing Ca9-22 cells, control HSC4 cells and Wnt-5b-overexpressing HSC4 cells. GAPDH expression was used as a loading control. *B,* MMP-10 knockdown into Periostin- and Wnt-5b-overexpressing cells. Cocktail of 3 different MMP-10 siRNAs was transiently transfected into Periostin-overexpressing Ca-9-22 cells and Wnt-5b-overexpressing HSC4 cells. A scrambled sequence that does not show significant homology to rat, mouse or human gene sequences was used as a control. After 48 h of transfection, MMP-10 protein level was examined by Western blot analysis with anti-MMP-10 antibody in MMP-10 siRNA transfected Periostin-overexpressing cells. ß-actin was used as a loading control. *C,* The invasiveness of MMP-10 siRNA transfected Periostin-overexpressing Ca-9-22 cells (left panel) and Wnt-5b-overexpressing HSC4 cells (right panel) was examined by *in vitro* invasion assay. After 18 h incubation of HSC4 cells and 24 h incubation of Ca9-22 cells, cells were fixed and the number of invaded cells was counted. The figure shows the stained lower side of the membrane where the cells penetrated (upper panel). Graphs show the number of invaded cells in knockdown and control cells (lower panel). The bars show the average values and SDs of three independent experiments. *Significantly different from control at *P*<0.01.

Our present findings revealed that MMP-10 is a common upregulated gene among Periostin, IFITM1, and Wnt-5b overexpression. We compared the expression of MMP-10 in Periostin, IFITM1, and Wnt-5b-overexpressing cells with that in control cells. MMP-10 was found to be upregulated by Periostin or Wnt-5b (Figure 5A), but not by IFITM1 (data not shown). In our microarray analysis, 17.4 fold increase of MMP-10 expression was observed in Periostin-overexpressing HSC2 cells and 5.8 fold increase of MMP-10 expression was observed in Wnt-5b-overexpressing HSC4 cells. Therefore, we examined the potential role of MMP-10 in Periostin and Wnt-5b-promoted invasion in HNSCC. We examined the effect of MMP-10 knockdown on the invasion in Periostin-overexpressing Ca9-22 cells and in Wnt-5b-overexpressing HSC4 cells. Expression of MMP-10 protein was reduced by MMP-10 knockdown in Periostin- and Wnt-5b-overexpressing cells (Figure 5B). Interestingly, MMP-10 knockdown significantly suppressed the Periostin- and Wnt-5b-promoted invasion (Figure 5C), indicating that MMP-10 may play a role in the invasiveness driven by Periostin- and Wnt-5b-overexpression in HNSCC. We also examined MMP-10 knockdown in Periostin-overxpressiong HSC2 cells (Figure S4). In similar to Periostin-overxpressiong Ca9-22 cells, MMP-10 siRNA suppressed the invasive capacities in Periostin-overxpressiong HSC2 cells. In addition, MMP-10 knockdown significantly inhibited the invasion in control Ca9-22 cells, while MMP-10 knockdown slightly inhibited the invasion in control HSC2 and HSC4 cells (Figure 5C and Figure S4). In Ca9-22 cells, high level of MMP-10 expression was observed, but not in HSC2 and HSC4 cells (Figure 4A). As Ca9-22 cells showed endogenous MMP-10 expression at higher levels, we thought that MMP-10 siRNA suppressed invasive capacities through downregulation of endogenous MMP-10 expression in Ca9-22 cells. The level of suppression by MMP-10 knockdown was most likely dependent on the expression level of MMP-10.

### MMP-10-promoted invasion is associated with p38 inhibition

**Figure 6:**
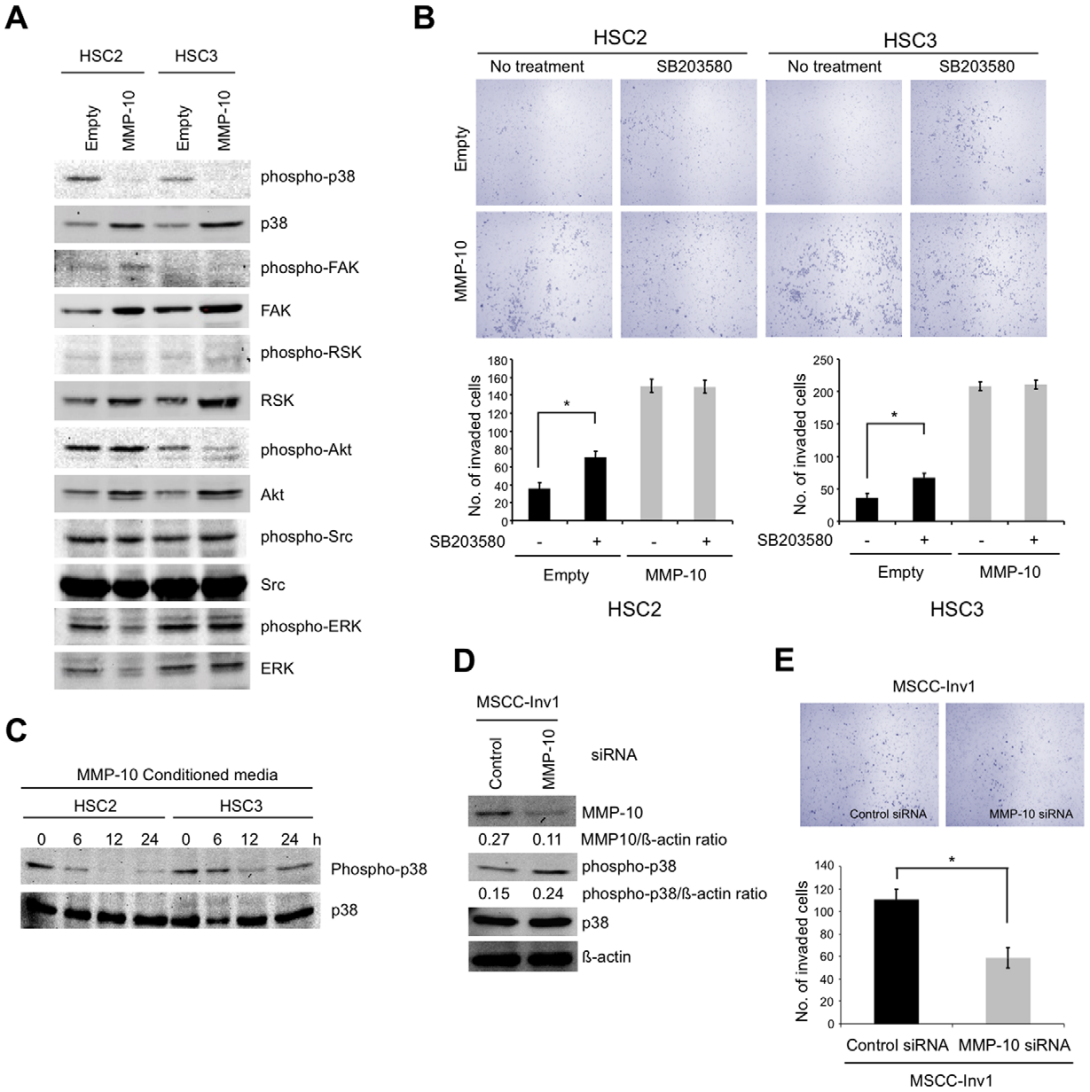
p38 inhibition is involved in promoting invasion by MMP-10. *A,* p38 inhibition was noted in MMP-10 overexpressing cells. Levels of total and phosphorylated forms of p38, FAK, RSK, Akt, Src, and ERK in control and MMP-10 overexpresing cells by western blotting. *B,* Invasion of control and MMP-10 overexpressing cells after treatment by p38 inhibitor. The invasiveness of was examined by *in vitro* invasion assay in empty vector transfected cells and MMP-10 overexpressing cells with SB203580 treatment. Empty vector transfected cells were used as a control. After 12 h incubation, cells were fixed and the number of invaded cells was counted. The figure shows the stained lower side of the membrane where the cells penetrated (upper panel). Graphs show the number of invaded cells (lower panel). The bars show the average values and SDs of three independent experiments. *Significantly different from empty vector transfected cells without SB203580 treatment at *P*<0.01. *C,* p38 activity after treatment with conditioned media from MMP-10 overexpressing cells. Conditioned media from MMP-10 overexpressing HSC2 and HSC3 cells was collected after 48 h of plating. After adding conditioned media for 0, 6, 12 and 24 h, HSC2 or HSC3 cells were collected. Expression of phospho-p38 and p38 was examined by Western blot analysis. *D,* MMP-10 siRNA upregulates p38 activity in HNSCC cells. Cocktail of 3 different MMP-10 siRNAs was transiently transfected into MSCC-Inv1 cells. A scrambled sequence that does not show significant homology to rat, mouse or human gene sequences was used as a control. After 48 h of transfection, expression of MMP-10 protein was examined by Western blotting. Levels of total and phosphorylated forms of p38 were also examined by Western blotting. β-actin expression was used as a loading control. *E,* Suppression of invasion by MMP-10 knockdown in MSCC-Inv1 cells. The invasiveness was examined by *in vitro* invasion assay. After 6 h incubation, cells were fixed and the number of invaded cells was counted. The figure shows the stained lower side of the membrane where the cells penetrated (upper panel). The graph shows the number of invaded cells (lower panel). The bars show the average values and SDs of three independent experiments. *Significantly different from control at *P*<0.01.

In order to know the mechanism of MMP-10 promoted invasion, we observed the activity of cell signaling molecules such as p38, FAK, RSK, Akt, Src, and ERK by western blotting using phosphorylation specific antibody in control and MMP-10 overexpressing cells. Among these molecules, p38 was inactivated by MMP-10 overexpression (Figure 6A). To further confirm the involvement of p38 inhibition in MMP-10-promoted invasion, the invasive ability of control and MMP-10-overexpressing cells after treatment with p38 inhibitor, SB203580 was examined. Interestingly, SB203580 treatment significantly promoted the invasion of control cells with p38 activity (Figure 6B). On the other hand, SB203580 treatment did not promote the invasion in MMP-10 overexpressing cells without p38 activity. In addition, we examined if a p38 inhibitor rescued the block of invasion induced by MMP10 knockdown in MMP-10 overexpressing HSC2 and HSC3 cells. MMP-10 knockdown in MMP-10-overexpressing cells promoted the invasive capacity after treatment with p38 inhibitor (Figure S5). However, p38 inhibitor did not fully rescue the invasive capacity suppressed by MMP-10 siRNA (Figure S5). Moreover, we confirmed that addition of conditioned media from MMP-10-overexpressing cells inhibited p38 activity in HSC2 and HSC3 cells (Figure 6C). We also examined the p38 activity and the invasion by MMP-10 knockdown in MSCC-Inv1 cells. In MSCC-Inv1 cells, MMP-10 knockdown slightly upregulated p38 activity (Figure 6D) and inhibited the invasive activity (Figure 6E).

## Discussion

Invasion and metastasis are the major problem and the greatest obstacle for the treatment of HNSCC. Therefore, it is important to identify novel molecules involved in the invasion and metastasis of HNSCC. We identified several invasion related genes by comparing the gene expression profiles between parent cells and a highly invasive clone [8]. Periostin and IFITM1 were identified as the highest genes in a highly invasive clone and their involvement in invasion were confirmed by *in vitro* and *in vivo* studies [8], [9]. Here, we demonstrated that Wnt-5b promoted the invasion of HNSCC cells (Figure 1). So far, there is only one report that Wnt-5b is upregulated in cancer cell lines and little is known about the role of Wnt-5b in cancer [16]. It has previously been shown that Wnt-5a is involved in the invasion of various cancers including melanoma, breast cancer, gastric cancer, pancreatic cancer and osteosarcoma [17]–[23]. As human Wnt-5b protein is 80% identical to human Wnt-5a protein, Wnt-5b may be involved in the migration and invasion of HNSCC in a similar manner.

In this study, we tried to identify the common target genes induced by Periostin, IFITM1 and Wnt-5b by using microarray data sets. Several molecules were identified as common upregulated genes by Periostin, IFITM1, and Wnt-5b overexpression (Table S5). Among them, we focused on the roles of MMP-10 in the invasion of HNSCC. MMPs belong to the metzincin superfamily of Zn-dependent proteinases [11]. All MMPs share the pro-domain and the catalytic domains, and act on broad spectrum of ECM components [24]. They are classified into secreted (soluble) and membrane-anchored MMPs and they are all synthesized as pro-enzymes, which require activation either extracellularly or intracellularly [25]. The soluble MMPs including collagenase, stromelysins, gelatinases, matrilysin, and others show broader accessibility to ECM and have a role in tissue remodeling events [26]. It seems necessary to know the types of MMPs that specifically facilitate invasion and metastasis in HNSCC. So far, MMP-2, MT1-MMP and MMP-9 are implicated in the invasion and metastasis of HNSCC [12], [13]. However, little is known about involvement of MMP-10 in the invasion and metastasis of HNSCC. In this study, we demonstrated that MMP-10 has the ability to promote the invasion of HNSCC cells *in vitro* (Figure 4). Indeed, high expression of MMP-10 was frequently observed in HNSCC cases and was well correlated with malignant behaviors such as high invasiveness, advanced stage and metastasis (Figure 3). Moreover, HNSCC cases with high expression of MMP-10 tended to show poor prognosis (Figure 3C). Our findings are supported by a recent paper showing that PKCι–Par6α–Rac1 signaling axis promoted anchorage-independent growth and invasion of non small cell lung carcinoma (NSCLC) cells through induction of MMP-10 expression [27]. In fact, MMP-10 expression is elevated in NSCLC tissues and NSCLC patients whose tumors express high MMP-10 levels exhibited significantly worse survival than those whose tumors express low MMP-10 [27], [28].

MMP-10/stromelysin-2 constitutes a group with MMP-3/stromelysin-1. Although MMP-10 and MMP-3 are similar in their amino acid sequence and substrate specificity, differential patterns of expression on normal and transformed cells are different between MMP-3 and MMP-10 [29]–[32]. In our analysis, MMP-3 was not upregulated by Periostin, IFITM1, and Wnt-5b overexpression (data not shown). MMP-10 degrades various components of the extracellular matrix and is considered as collagenase-related connective tissue-degrading metalloproteinases. The ability of MMP-10 to promote neoplasia has been inferred to its secretion by peritumoral cells in response to the presence of signals provided by the tumor cells [33]. In our analysis, HNSCC cells themselves secreted MMP-10 in conditioned media and promoted their invasiveness. The degradation of collagenase-related connective tissue may be involved in promoting invasiveness of HNSCC cells. Van Themsche et al. also clearly showed that lymphoma cells could secrete MMP-10, notably following exposure to cytokines such as IL-4, IL-6, and IL-13 [34]. Interestingly, MMP-10 is also upregulated in tongue cancer by comparing genome-wide transcriptomic profiles between 53 primary tongue cancer and 22 matching normal tissues [35]. Moreover, other group showed that MMP-10 is a potential oral cancer marker [36].

In this study, we found upregulation of MMP-10 by Periostin or Wnt-5b overexpression, but not by IFITM1 (Figure 5A). Interestingly, MMP-10 knockdown inhibited the invasion promoted by Periostin or Wnt-5b overexpression (Figure 5C and Figure S4). These findings indicate that MMP-10 may play an important role in Periostin- or Wnt-5b-mediated invasion. In Figure 1B, Wnt-5b knockdown suppressed invasion in HSC2 cells in spite of low expression of MMP-10. We found that knockdown of MMP-10 in HSC2 cells suppressed the invasion (67% of number of invaded cells in comparison with control) (Figure S4). Therefore, we suggest that Wnt-5b knockdown-suppressed invasion in HSC2 may be due to in part by MMP10 knockdown. Unknown factors may influence on the regulation of MMP-10. We still do not know the mechanism of upregulation of MMP-10 by Wnt-5b overexpression. It has recently been reported that Periostin was frequently overexpressed in various types of human cancers [37]. We already demonstrated that interaction between Periostin and integrins has a role in promoting invasion of HNSCC by triggering the intracellular signaling and activating certain genes that are involved in invasion [8], [38]. In a previous report, Periostin and MMP-10 were selectively upregulated in human osteoblasts in comparison with human fibroblasts by microarray analysis [39]. As integrins can regulate the expression and activation of MMPs and can guide them to their targets by simultaneous binding of MMPs and ECM molecules [40], the interaction between integrin and Periostin may induce MMP-10 expression. In addition, some MMPs including MMP-1, MMP-2 and MMP-13 are involved in Wnt-5a mediated invasion of cancer cells [19], [21], [41], [42]. As human Wnt-5b protein is 80% identical to human Wnt-5a protein, MMP-10 may play an important role in Wnt-5b-mediated invasion of HNSCC cells. As both Periostin and Wnt-5b are secreted protein, MMP-10 may be transcriptionally upregulated by Periostin- or Wnt-5b-mediated intracellular signaling. It has recently been reported that MMP-10 is induced by TGF-ß mediated by activation of MEF2A and downregulation of class IIa HDACs [43]. We checked the microarray data of control vs Periostin-overexpressing cells and control vs Wnt-5b-overexpressing cells. Interestingly, MEF2A was upregulated 2.5-fold by Wnt-5b overexpression (Data S1), suggesting that upregulation of MEF2A by Wnt-5b overexpression may promote transcriptional activation of MMP-10. In Periostin overexpression cells, upregulation of MEF2A was not observed (data not shown). Thus, MMP-10 may be transcriptionally upregulated through intracellular signaling. To clarify the detailed mechanism of MMP-10 regulation by Periostin or Wnt-5b in HNSCC cells, further experiments are required.

In this study, we found that p38 MAPK activity was inhibited by MMP-10 overexpression, and that the inhibition of p38 MAPK was associated with MMP-10-promoted invasion. Moreover, we examined if a p38 inhibitor rescued the block of invasion induced by MMP10 knockdown in MMP-10 overexpressing HSC2 and HSC3 cells. p38 inhibitor treatment slightly increased the invasion in MMP-10 overexpressing HSC2 and HSC3 cells (Figure S5). However, the invasive activity was not fully recued by suppression of p38 activity. In addition, we found p38 activity was upregulated by MMP-10 knockdown in MSCC-Inv1 cells. These results indicate that suppression of p38 activity may be partially involved in the invasion of HNSCC. This speculation is supported by the previous findings that i) the inhibition of cancer cell invasion was mediated by the activation of p38 MAPK pathway [44], and ii) p38 activation was shown to suppress the metastatic colonization in ovarian cancer [45]. However, the mechanism of promoting invasion by suppression of p38 activity is still unclear. As shown in Figure 6C, addition of conditioned media from MMP-10 overexpressing cells suppressed p38 activity, indicating that p38 activity is inhibited by secreted MMP-10 through intracellular signaling. We also examined the p38 activity in Periostin or Wnt-5b overexpressing cells (Figure S6). Wnt-5b overexpressing suppressed p38 activity, but Periostin overexpression did not. Suppression of p38 activity through upregulation of MMP-10 by Periostin may be required for another factor.

In summary, we have demonstrated that MMP-10 promotes invasion in HNSCC and that MMP-10 mediates the Periostin- and Wnt-5b-induced invasion. Indeed, the inhibition of p38 MAPK was partially involved in MMP-10 driven invasion in HNSCC cells. These findings provide new insights into the roles of MMP-10 in promoting invasion in HNSCC and indicate that MMP-10 can be a valuable marker for predicting the invasion and metastasis in HNSCC patients.

## Materials and Methods

### Cell lines and culture conditions

HNSCC cell lines (HCS2, HSC3, HSC4, Ca-9-22, Ho-1-N-1, and Ho-1-U-1) were provided by the Japanese Collection of Research Bioresources Cell Bank. These cells were maintained in RPMI 1640 (Nacalai Tesque, Inc., Kyoto, Japan) supplemented with 10% heat-inactivated fetal bovine serum (Gibco) and 100 units/ml penicillin-streptomycin (Gibco) under the condition of 5% CO_2_ in air at 37°C. For proliferation assay, 5000 cells were plated on 24-well plate and the cells were allowed to grow and expand. The cells were then trypsinized and counted at 0, 2, 4, and 6 days by using cell counter (Coulter Z1, Beckman-Coulter).

### RT-PCR

Using RNeasy mini kit (Qiagen), total RNA from cultures of confluent cells was isolated. These isolates were quantified and their purity was evaluated by spectrophotometer. The cDNA was synthesized from 1 µg of total RNA according to ReverTra Dash (Toyobo Biochemicals, Tokyo, Japan). We used the following primers; human Wnt-5b, 5′-cgggagcgagagaagaact-3′ (forward) and 5′-tacacctgacgaagcagcac-3′ (reveres: product size 505 bp), human Wnt-5a, 5′-ggctggaagtgcaatgtctt-3′ (forward) and 5′-ccgatgtactgcatgtggtc-3′ (reveres: product size 234 bp), human MMP-10, 5′-ggctctttcactcagccaac-3′ (forward) and 5′-tcccgaaggaacagattttg-3′ (reveres: product size 176 bp) and human glyceraldahyde-3-phosphate dehydrogenase (GAPDH) 5′-tccaccaccctgttgctgta-3′ (forward) and 5′-accacagtccatgccatcac-3′ (reverse: product size: 450 bp). Aliquots of total cDNA were amplified with 1.25 units of rTaq-DNA polymerase (Qiagen) and this amplification was done in a thermal cycler (MyCyler, Bio-Rad, Richmond, CA) for 30 cycles after initial 30 seconds of denaturation at 94°C, annealing for 30 seconds at 60°C, and extension for 1 minute at 72°C in all primers used. The amplification reaction products were resolved on 1.2 % agarose/TAE gels (Nacalai Tesque, Kyoto, Japan), electrophoresed at 100 mV, and then finally visualized by using ethidium bromide.

### Western blot analysis

Western blotting was carried out as previously described [8]. The protein concentrations were measured by Bradford protein assay (Bio-Rad). Twenty µg of protein was subjected to 10 % polyacrylamide gel electrophoresis, followed by electroblotting onto a nitrocellulose filter. For detection of the immunocomplex, the ECL western blotting detection system (Amersham) was used. Anti-MMP-10 monoclonal antibody (Novocastra), anti-FLAG monoclonal antibody (Sigma) and anti-ß-actin monoclonal antibody (Sigma), phospho-p38 (Thr180/Tyr182) monoclonal antibody (Cell Signaling Technology), phospho-FAK (Tyr576/Tyr577) monoclonal antibody (Cell Signaling Technology), phospho-RSK (Ser380) polyclonal antibody (Cell Signaling Technology), phospho-Akt (Ser473) monoclonal antibody (Cell Signaling Technology), phospho-Src (Tyr416) polyclonal antibody (Cell Signaling Technology), phospho-ERK monoclonal antibody (Santa Cruz Biotechnology. Inc), anti-p38 polyclonal antibody (Cell Signaling Technology), anti-FAK polyclonal antibody (Cell Signaling Technology), anti-RSK polyclonal antibody (Cell Signaling Technology), anti-Akt polyclonal antibody (Cell Signaling Technology), anti-Src polyclonal antibody (Cell Signaling Technology), and anti-ERK monoclonal antibody (Cell Signaling Technology) were used. Anti-Wnt-5b antibody was generated in rabbits by immunization with synthetic peptides corresponding to residue 254–269. For detection of phosphorylated proteins, membranes were blocked with 3% milk/TBS-T and incubated with phospho-specific antibodies overnight at 4°C. After washing in TBS-T, membranes were incubated with specific secondary antibodies, and the proteins were visualized as described earlier.

### Generation of Wnt-5b-overexpressing HNSCC cells

The Wnt-5b plasmid (pPGK2-Wnt-5b) or the empty vector was introduced into HSC4 cells, and the stable clones were obtained by G418 selection (500 µg/ml, Gibco) in the culture medium. We obtained pool and 4 stable clones. Cell transfection was performed using FuGENE 6 HD (Roche) according to the manufacturer's instruction.

### Silencing by siRNA

Wnt-5b siRNA (Wako, Osaka, Japan) is a 21-bp duplex oligoribonucleotides corresponding to the nucleotides of the human Wnt-5b mRNA sequences. The sense sequence is gaacuuugccaaaggaucaTT. Three MMP-10 siRNAs (Oligo ID, 1135A-C, B-Bridge International, Inc.) are 21-bp duplexes oligoribonucleotides corresponding to the nucleotides of the human MMP-10 mRNA sequences. The sense sequences are: #1; gaaguuaacagcagggacaTT, #2; gaguugagccuaagguugaTT, and #3; gugcaauaggugagagaauTT. Logarithmically growing cells were seeded at a density of 10^5^ cells per 6-cm dish and transfected with 5 nM Wnt-5b siRNA or 5 nM cocktail of 3 MMP-10 siRNAs using Oligofectamine (Invitrogen) according to manufacturer's instructions. Forty-eight hours after transfection, cells were used for *in vitro* invasion assay as described below. A scrambled sequence that does not show significant homology to rat, mouse or human gene sequences was used as a negative control. The efficiency of siRNA was checked by RT-PCR.

### *In vitro* invasion assay

This experiment was done as previously described [8]. Briefly, a 24-well cell culture insert with 8 µm pores (3097, Falcon, Becton Dickinson) was used. The membrane was coated by 20 µg of matrigel (Becton Dickinson), which is reconstituted basement membrane substance. The lower compartment contained 0.5 ml of serum-free medium. After trypsinization, 1.5×10^5^ cells were resuspended in 100 µl of serum-free medium and placed in the upper compartment of the cell culture insert for 6–24 hours. In an additional assay, p38 inhibitor (SB203580; sigma) was added to control and MMP-10 overexpressing cells after trypsinization and before plating the cells on the upper compartment of the insert. After incubation at 37°C, we fixed the cells that penetrate the membrane into the lower side with formalin and these cells were then stained by hematoxylin. The invasiveness of the cells was determined by counting the penetrating cells onto the lower side of the filter through the pores under a microscope at x100 magnification. We assayed 3 times and randomly selected 3 fields were counted for each assay.

### Wound healing assay

For the wounding healing experiment, cells were seeded on 6 well plates and cells were allowed to grow to complete confluence. Subsequently, a plastic pipette tip was used to scratch the cell monolayer to create a cleared area, and the wounded cell layer was washed with fresh medium to remove loose cells. Immediately following scratch wounding (0 h) and after incubation of cells at 37°C for 24 h, phase-contrast images (10× field) of the wound healing process were photographed digitally with an inverted microscope. The distance of the wound areas were measured on the images, set at 100% for 0 h, and the mean percentage of the total distances of the wound areas was calculated.

### Gene array analysis

The human focus array using the system containing 50000 genes probes was used for comparing the transcriptional profiles between Wnt-5b overexpressing HSC4 cells and control HSC4 cells. This array contains a broad range of genes derived from publicly available, well-annotated mRNA sequences. Total RNA was isolated from cultures of confluent cells using the RNeasy Mini Kit (Qiagen) according to the manufacturer's instructions. Preparations were quantified and their purity was determined by standard spectrophometric methods. Data were expressed as the average differences between the perfect match and mismatch probes for the HSC4 gene (Data S1).

### Tissue samples

Tissue samples of HNSCC were retrieved from the Surgical Pathology Registry of University of Palermo (Italy), University of Peradeniya and OMF unit, Kandy hospital (Sri Lanka), after approval by the Ethical Committee of each institution. Informed consent obtained from all patients was verbal for this study, and then signature was obtained from all patients. For immunohistochemical examination of MMP-10, 49 tissue samples of HNSCC were selected from the Surgical Pathology Registry of University of Palermo (Italy), and 67 tissue samples of HNSCC were selected from the pathological files of University of Peradeniya and OMF unit, Kandy hospital (Sri Lanka). Forty-nine Italian HNSCC cases (32 male and 17 female; average age is 63.3±12.6) were surgically resected from 1998 to 2003 before radiochemotherapy. Sixty-seven Sri Lankan HNSCC cases (42 male, 9 female and 16 unknown; average age is 50.2±13.2) were surgically resected from 1998 to 2004 before radiochemotherapy. Collectively, 116 HNSCC cases were used for evaluation of MMP-10 expression by immunohistochemical staining. Clinical information including age, location, tumor size, invasion pattern, metastasis, tumor stage, TNM classification, treatment and survival was gathered from surgical records of the patients (Data S2). Among 116 HNSCC cases, 63 cases showed metastasis to lymph node, and 2 cases showed metastasis to lymph node and other organs. Moreover, 42 Italian HNSCC cases were available for survival analysis. Survival time was calculated as the time interval between diagnosis and date of last information. For deceased cases, the date of last information was the date of death. For cases not known to be deceased, the date of last information was the date that the case was last known to be alive. All deceased cases died from HNSCC. Tissues were fixed in 10% buffered formalin and embedded in paraffin.

### Immunohistochemistry

Tumor tissues were fixed in 10% formalin, embedded in paraffin and cut into 4 µm thick. All HNSCC cases were classified according to invasion pattern into 4 grades; pattern I, pattern II, pattern III, and pattern IV [14]. For immunohistochemical staining, tissue sections were deparafinized in xylene, rehydrated in descending grades of ethanol. Endogenous peroxidase activity was blocked with methanol containing 0.3% H_2_O_2_ for 30 minutes. Antigen retrieval was done by microwaving using citrate phosphate buffer (pH 6.0), then the sections were incubated with the primary antibodies at 4°C overnight. Immunohistochemical staining was carried out by a monoclonal anti-MMP-10 antibody (Novocastra, 1∶300). For detection of the reaction after incubation with secondary antibodies, we used diaminobenzidine (DAKO). The sections were counterstained by haematoxylen, dehydrated in ascending grades of ethanol and finally the slides were mounted. MMP-10 expression was defined as high (over 10 % of the tumor cells showed strong or diffuse staining) and low (less than 10 % of the cells showed weak or no staining) by considering the percentage of positive cells and overall staining intensity.

### Generation of MMP-10-overexpressing cells

We cloned human MMP-10 into pBICEP2 (Sigma) using cDNA of Ho-1-N-1 cells. We transfected MMP-10 into HSC2 and HSC3 cells. Then, G418 (300 µg/ml, Gibco) was added to the culture medium after 48 h of transfection. After 2 weeks of G418 selection, we obtained the stable pool clones. Cell transfections were performed using FuGENE 6HD (Roche) according to the manufacture's instruction.

### Stromelysin zymography

To detect the activity of MMP-10, we used Mini Zymo Electrophoresis Kit (Life Laboratory Company, Yamagata, Japan), according to the manufacturer's instructions with some modifications. Briefly, cells were cultured for 48 h after plating. Then, conditioned media were collected and were concentrated using Amicon Ultra Centrifugal filter devices (Millipore). The conditioned media were mixed with SDS sample buffer provided with the kit and the mixtures were incubated for 15 min at room temperature. The samples were then subjected to gel electrophoresis. After washing, the gel was incubated for 40 h at 37°C in the enzymatic reaction buffer. Then, the gel was stained in Coomasie brilliant blue and destained in methanol/acetic acid solution with gentle agitation on a shaking plate. The activity of MMP-10 was identified by detection of clear bands in a background with a uniform staining.

### Statistical analysis

A *P* value <0.05 was required for assessing the significance. Correlation between variables was estimated using Fisher's exact test. Survival estimates were calculated by the Cox proportional hazards method with one-month intervals. Statistical significance based on Cox proportional hazards was determined using 95% confidence intervals. The appropriateness of the Cox proportional hazards model was assessed graphically.

## Supporting Information

Data S1 Data of microarray analysis for comparing the gene expression profiles between empty vector transfected HSC4 cells and Wnt-5b transfected HSC4 cells.
(XLS)

Data S2 Summary of immunohistochemical staining including information of HNSCC patients, clinico-pathological findings and MMP-10 expression.
(XLS)

Figure S1 **Antibody specificity of immunohistochemical staining of MMP-10.** To demonstrate the antibody specificity of immunohistochemical staining of MMP-10, we performed immunohistochemical staining without secondary antibody as a negative control. We also performed immunohistochemical staining without primary antibody as a negative control. Two representative case of immuno-expression. Scale bar is shown in each picture.
(TIFF)

Figure S2 **Diagrammatic illustration of invasion pattern** (Jacobson et al. [14]). Pattern I: solid sheets with pushing border, Pattern II: comparatively large tumor islands, Pattern III: thin strands of tumor cells as well as small tumor islands, and Pattern IV: scattered individual tumor cells.
(TIFF)

Figure S3 **The efficiency of MMP-10 siRNA.** Four different MMP-10 siRNAs (#1, #2, #3 and cocktail) were transiently transfected into Ca9-22 cells. A scrambled sequence that does not show significant homology to rat, mouse or human gene sequences was used as a control. After 48 h from transfection, MMP-10 mRNA was examined by RT-PCR. GAPDH was used as a loading control.
(TIFF)

Figure S4 **MMP-10 knockdown into Periostin-overexpressing cells.** Cocktail of 3 different MMP-10 siRNAs was transiently transfected into Periostin-overexpressing HSC2 cells. A scrambled sequence that does not show significant homology to rat, mouse or human gene sequences was used as a control. After 48 h of transfection, the invasiveness of MMP-10 siRNA transfected Periostin-overexpressing HSC2 cells was examined by *in vitro* invasion assay. After 14 h incubation of HSC2, cells were fixed and the number of invaded cells was counted. Graphs show the number of invaded cells in knockdown and control cells. The bars show the average values and SDs of three independent experiments. *Significantly different from control at P<0.01.
(TIFF)

Figure S5 **SB203580 treatment in MMP-10 siRNA treated MMP-10-overexpressing cells.** MMP-10 siRNA was transfected into MMP-10-overexpressing HSC2 cells and MMP-10-overexpressing HSC3 cells. Cocktail of 3 different MMP-10 siRNAs was transiently transfected into MMP-10-overexpressing cells. A scrambled sequence that does not show significant homology to rat, mouse or human gene sequences was used as a control. After 48 h of transfection, cells were used for *in vitro* invasion assay with or without p38 inhibitor, SB203580. After 12 h incubation, cells were fixed and the number of invaded cells was counted. The figure shows the stained lower side of the membrane where the cells penetrated (upper panel). The graph shows the number of invaded cells (lower panel). The bars show the average values and SDs of three independent experiments. Scale bar is shown.
(TIFF)

Figure S6 **p38 activity in Periostin-overexpressing Ca9-22 and HSC4 cells and Wnt-5b-overexpressing HSC4 cells.** Expression of phospho-p38 and p38 was examined by Western blot analysis. ß-actin expression was used as a loading control.
(TIFF)

Table S1 Upregulated and downregulated genes in Wnt-5b-overexpressing cells.
(TIFF)

Table S2 Common up-regulated gene by Periostin and IFITM1 overexpression in HNSCC cells.
(TIFF)

Table S3 Common up-regulated gene by Periostin and Wnt5B overexpression in HNSCC cells.
(TIFF)

Table S4 Common up-regulated gene by IFITM1 and Wnt5B overexpression in HNSCC cells.
(PDF)

Table S5 Common up-regulated gene by Periostin, IFITM1 and Wnt-5b overexpression.
(TIFF)
