# Correction: MMP-10/Stromelysin-2 Promotes Invasion of Head and Neck Cancer

**DOI:** 10.1371/annotation/ef18e199-e66d-43b9-97e0-96d1ab149193

**Published:** 2012-02-01

**Authors:** Elsayed Mohamed Deraz, Yasusei Kudo, Maki Yoshida, Mariko Obayashi, Takaaki Tsunematsu, Hirotaka Tani, Samadarani B. S. M. Siriwardena, Mohammad Reza Keikhaee, Guangying Qi, Shinji Iizuka, Ikuko Ogawa, Giuseppina Campisi, Lorenzo Lo Muzio, Yoshimitsu Abiko, Akira Kikuchi, Takashi Takata

There was a spelling error in the eighth author's name. The correct spelling is Mohammad Reza Keikhaee. 

