# Supplementary figures and images for: MMP-10/Stromelysin-2 Promotes Invasion of Head and Neck Cancer
Source: PLoS One. 2011 Oct 5;6(10):e25438. doi: 10.1371/journal.pone.0025438 (PMC3187776; doi:10.1371/journal.pone.0025438)

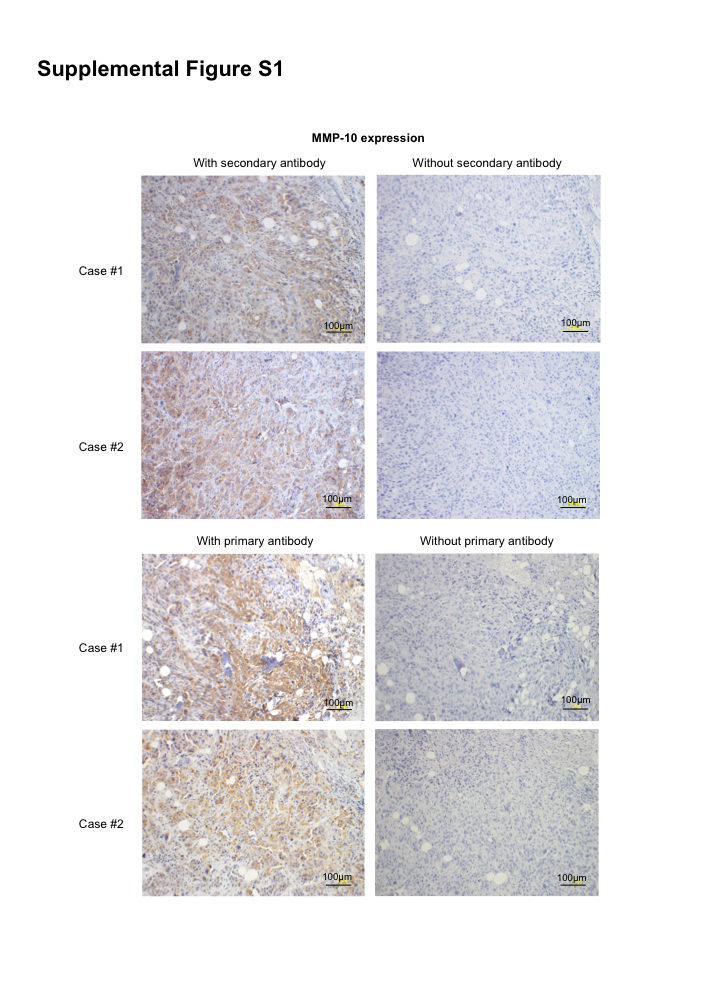

Supplement: Figure S1 — Antibody specificity of immunohistochemical staining of MMP-10. To demonstrate the antibody specificity of immunohistochemical staining of MMP-10, we performed immunohistochemical staining without secondary antibody as a negative control. We also performed immunohistochemical staining without primary antibody as a negative control. Two representative case of immuno-expression. Scale bar is shown in each picture. (TIFF) [file pone.0025438.s003.tif]

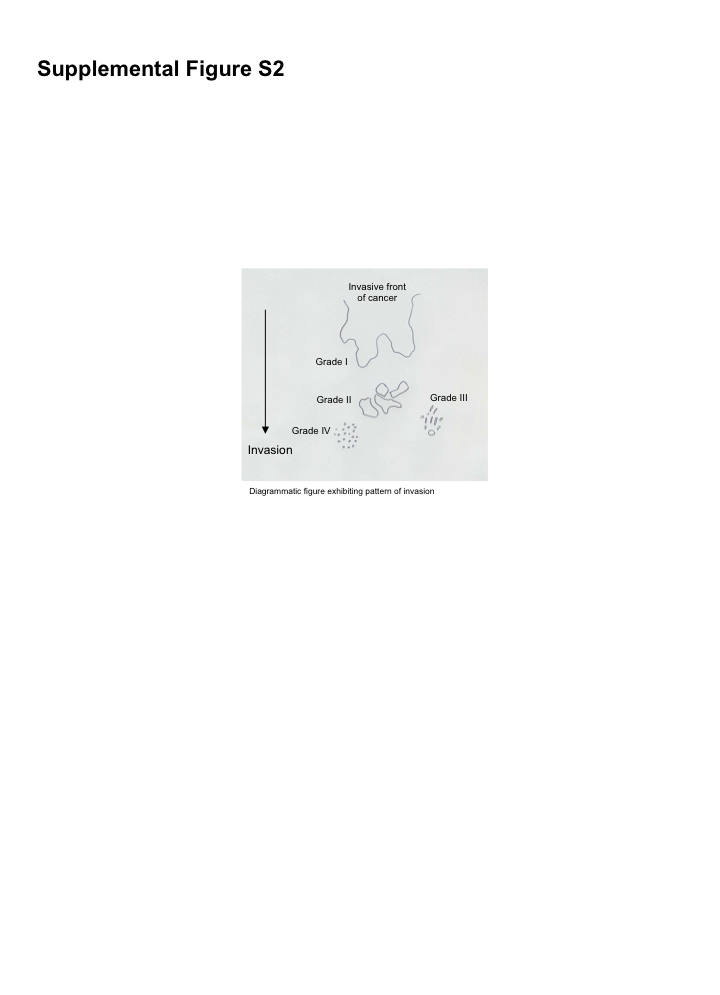

Supplement: Figure S2 — Diagrammatic illustration of invasion pattern (Jacobson et al. [14]). Pattern I: solid sheets with pushing border, Pattern II: comparatively large tumor islands, Pattern III: thin strands of tumor cells as well as small tumor islands, and Pattern IV: scattered individual tumor cells. (TIFF) [file pone.0025438.s004.tif]

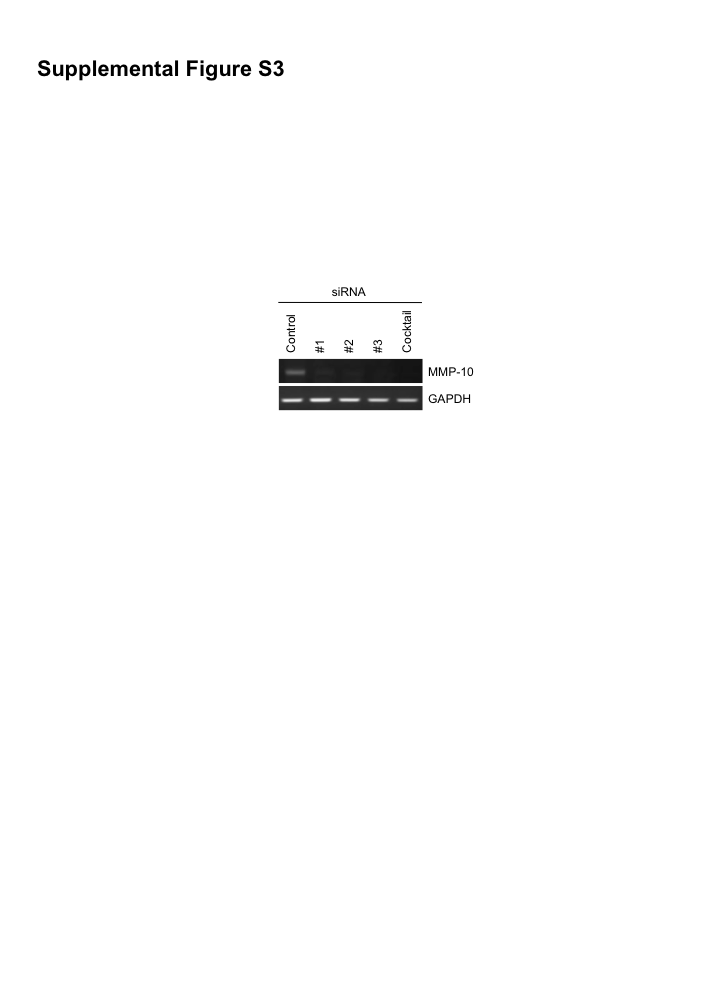

Supplement: Figure S3 — The efficiency of MMP-10 siRNA. Four different MMP-10 siRNAs (#1, #2, #3 and cocktail) were transiently transfected into Ca9-22 cells. A scrambled sequence that does not show significant homology to rat, mouse or human gene sequences was used as a control. After 48 h from transfection, MMP-10 mRNA was examined by RT-PCR. GAPDH was used as a loading control. (TIFF) [file pone.0025438.s005.tif]

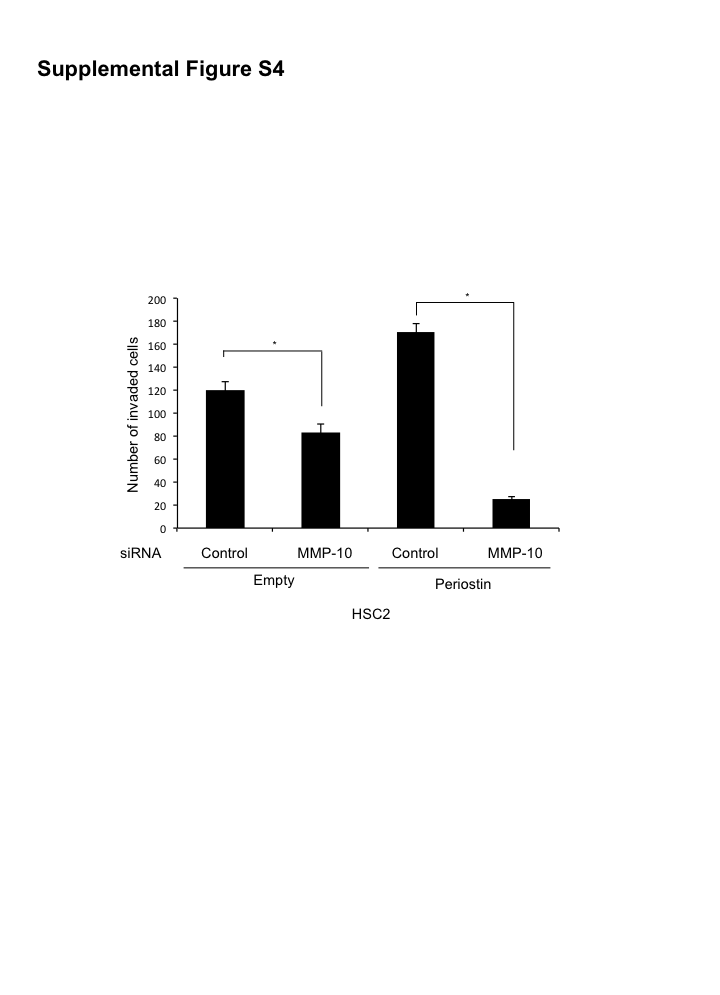

Supplement: Figure S4 — MMP-10 knockdown into Periostin-overexpressing cells. Cocktail of 3 different MMP-10 siRNAs was transiently transfected into Periostin-overexpressing HSC2 cells. A scrambled sequence that does not show significant homology to rat, mouse or human gene sequences was used as a control. After 48 h of transfection, the invasiveness of MMP-10 siRNA transfected Periostin-overexpressing HSC2 cells was examined by in vitro invasion assay. After 14 h incubation of HSC2, cells were fixed and the number of invaded cells was counted. Graphs show the number of invaded cells in knockdown and control cells. The bars show the average values and SDs of three independent experiments. *Significantly different from control at P<0.01. (TIFF) [file pone.0025438.s006.tif]

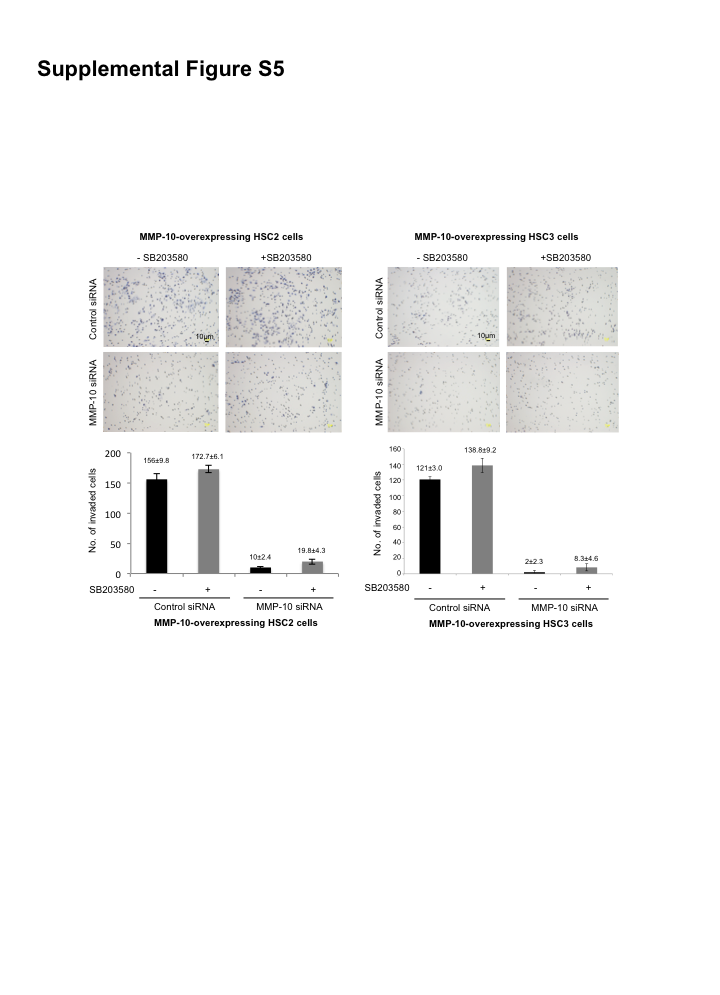

Supplement: Figure S5 — SB203580 treatment in MMP-10 siRNA treated MMP-10-overexpressing cells. MMP-10 siRNA was transfected into MMP-10-overexpressing HSC2 cells and MMP-10-overexpressing HSC3 cells. Cocktail of 3 different MMP-10 siRNAs was transiently transfected into MMP-10-overexpressing cells. A scrambled sequence that does not show significant homology to rat, mouse or human gene sequences was used as a control. After 48 h of transfection, cells were used for in vitro invasion assay with or without p38 inhibitor, SB203580. After 12 h incubation, cells were fixed and the number of invaded cells was counted. The figure shows the stained lower side of the membrane where the cells penetrated (upper panel). The graph shows the number of invaded cells (lower panel). The bars show the average values and SDs of three independent experiments. Scale bar is shown. (TIFF) [file pone.0025438.s007.tif]

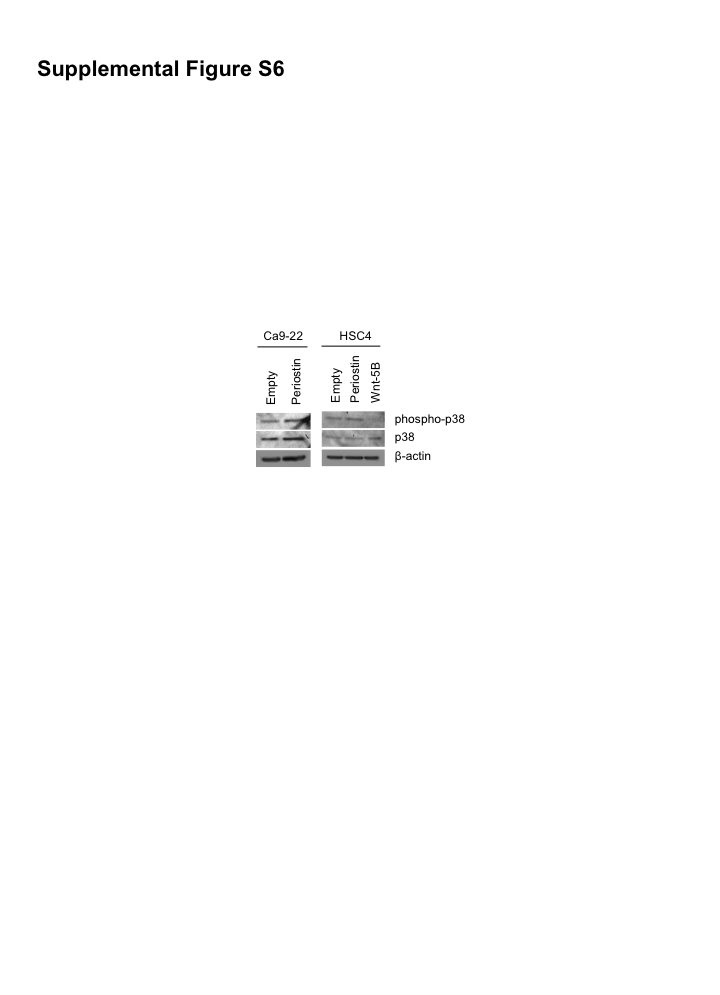

Supplement: Figure S6 — p38 activity in Periostin-overexpressing Ca9-22 and HSC4 cells and Wnt-5b-overexpressing HSC4 cells. Expression of phospho-p38 and p38 was examined by Western blot analysis. ß-actin expression was used as a loading control. (TIFF) [file pone.0025438.s008.tif]

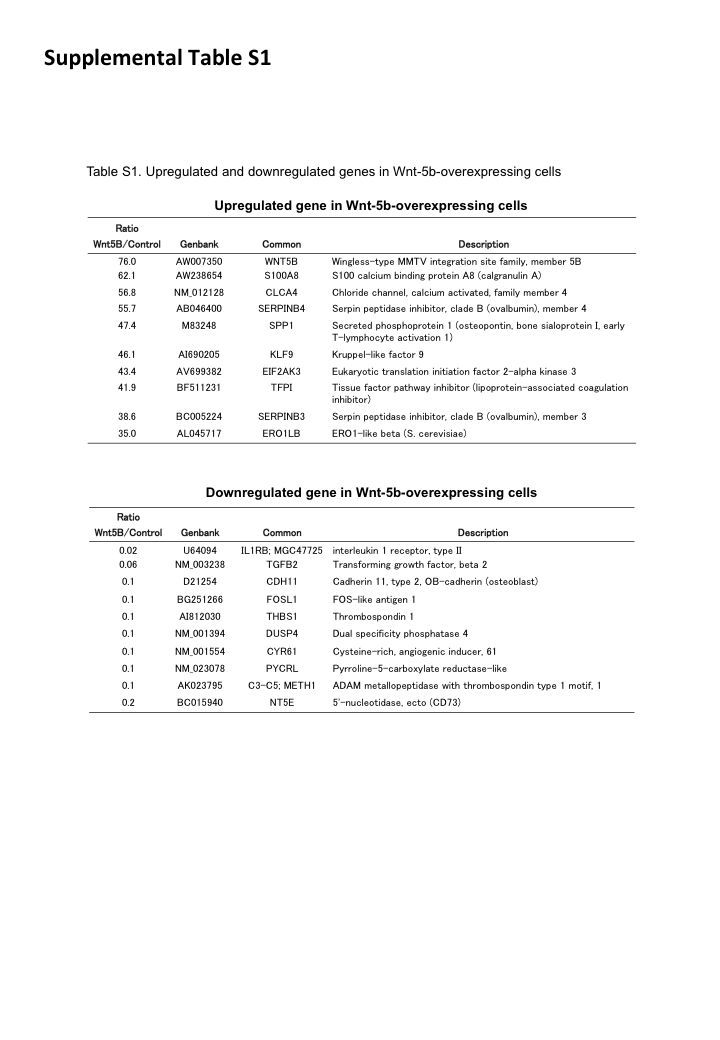

Supplement: Table S1 — Upregulated and downregulated genes in Wnt-5b-overexpressing cells. (TIFF) [file pone.0025438.s009.tif]

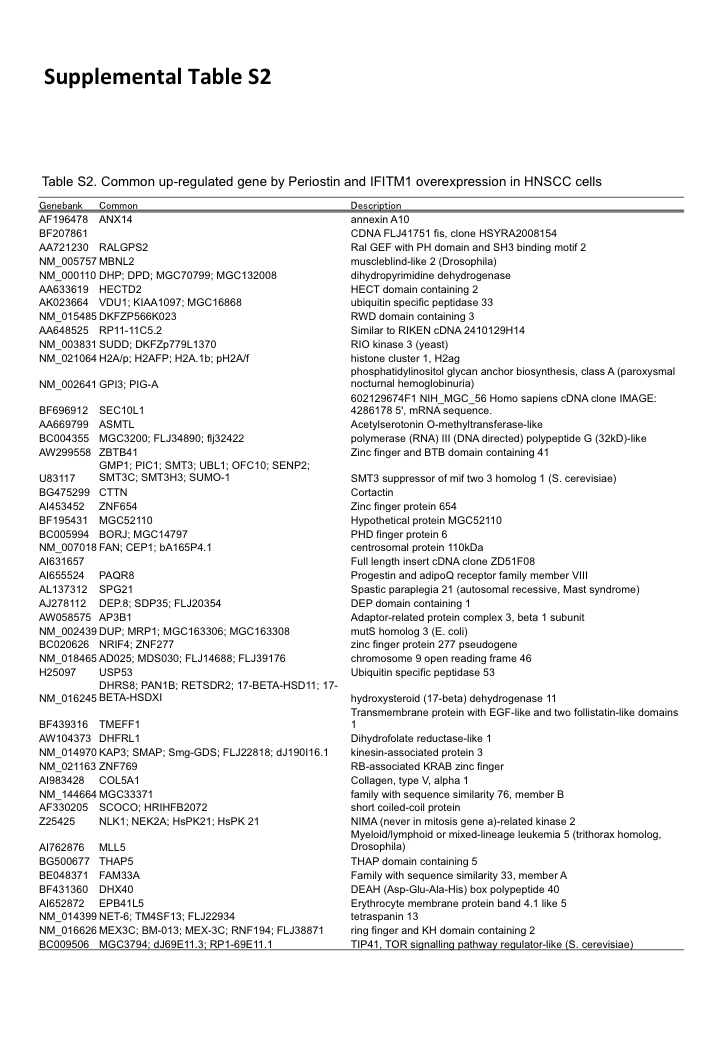

Supplement: Table S2 — Common up-regulated gene by Periostin and IFITM1 overexpression in HNSCC cells. (TIFF) [file pone.0025438.s010.tif]

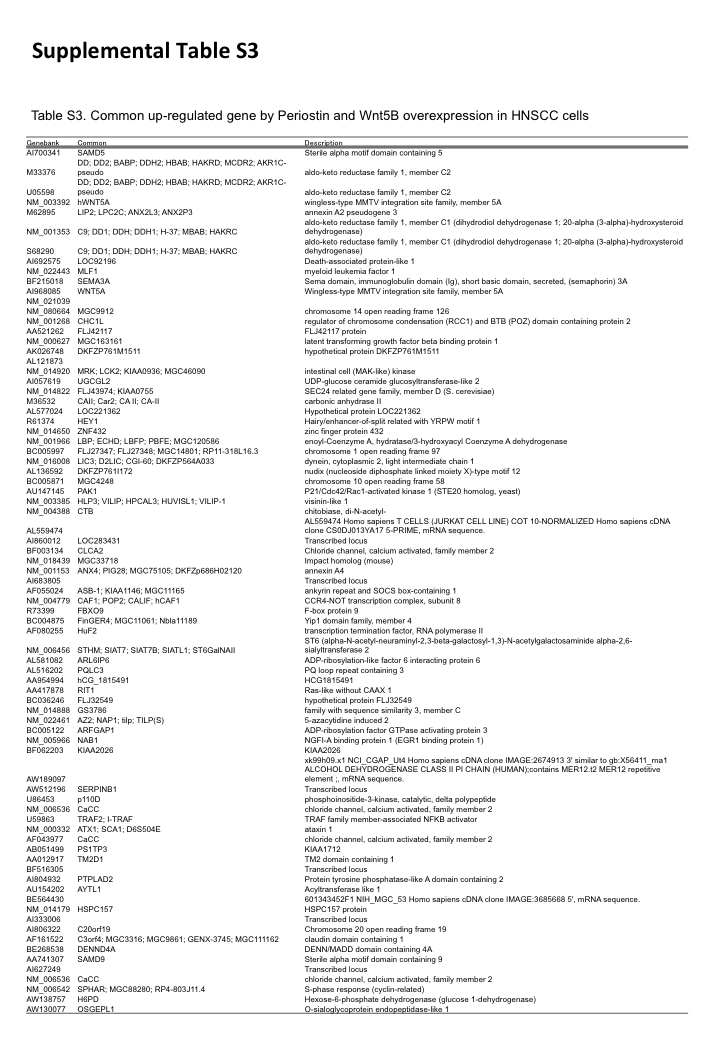

Supplement: Table S3 — Common up-regulated gene by Periostin and Wnt5B overexpression in HNSCC cells. (TIFF) [file pone.0025438.s011.tif]

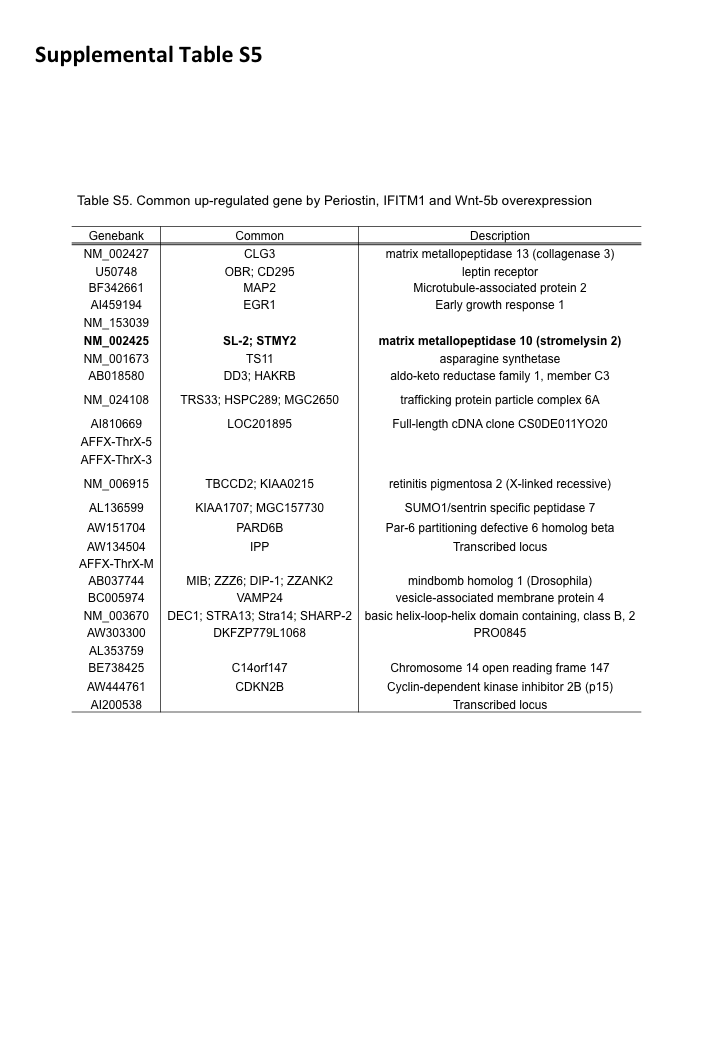

Supplement: Table S5 — Common up-regulated gene by Periostin, IFITM1 and Wnt-5b overexpression. (TIFF) [file pone.0025438.s013.tif]
