# Supplementary material for: MMP-10/Stromelysin-2 Promotes Invasion of Head and Neck Cancer
Source: PLoS One. 2011 Oct 5;6(10):e25438. doi: 10.1371/journal.pone.0025438 (PMC3187776; doi:10.1371/journal.pone.0025438)
Supplement: Table S4 — Common up-regulated gene by IFITM1 and Wnt5B overexpression in HNSCC cells. (PDF) [file pone.0025438.s012.pdf]

Supplemental Table S4

Supplemental Table S4. Common up-regulated gene by IFITM1 and Wnt5B overexpression in HNSCC cells

Genebank	Common	Description
AA053711	EDIL3	EGF-like repeats and discoidin I-like domains 3
NM_024722	FLJ13322; FLJ90623; HMFT0700	acyl-Coenzyme A binding domain containing 4
NM_002426	HME; MME; MGC138506	matrix metallopeptidase 12 (macrophage elastase)
NM_004585	RIG1; TIG3; HRASLS4; MGC8906	retinoic acid receptor responder (tazarotene induced) 3
BC020718	FI; IF; KAF; C3b-INA; factor I	complement factor I
NM_002064	GRX; GRX1; MGC117407	glutaredoxin (thioltransferase)
NM_021140	MGC141941; bA386N14.2; DKFZp686A03225	ubiquitously transcribed tetratricopeptide repeat, X chromosome
NM_001964	TIS8; AT225; G0S30; NGFI-A; ZNF225; KROX-24; ZIF-268	early growth response 1
NM_004354	CCNG2	cyclin G2
NM_001393	MGC126355; MGC126356	extracellular matrix protein 2, female organ and adipocyte specific
AV757675	OPTN	Optineurin
NM_002800	LMP2; RING12; MGC70470	proteasome (prosome, macropain) subunit, beta type, 9 (large multifunctional peptidase 2)
NM_017938	FLJ20716; RP3-525N14.6	family with sequence similarity 70, member A
U36189	P311; PTZ17; D4S114; PRO1873	chromosome 5 open reading frame 13
NM_002053	GBP1	guanylate binding protein 1, interferon-inducible, 67kDa
NM_000240	MAOA	monoamine oxidase A
AW006123	FBXO32	F-box protein 32
NM_004120	GBP2	guanylate binding protein 2, interferon-inducible
BF509371		Transcribed locus
AV733950	EGR1	Early growth response 1
AF141347	TUBA3; FLJ25113; B-ALPHA-1	tubulin, alpha 1a
AW303375	CCDC80	Coiled-coil domain containing 80
L49506	CCNG2	cyclin G2
BC040593	LOC340340	hypothetical protein LOC340340
AA812232	TXNIP	Thioredoxin interacting protein
NM_005715	2OST	uronyl-2-sulfotransferase
AF162769	GRX; GRX1; MGC117407	glutaredoxin (thioltransferase)
NM_016061	CGI-127	yippee-like 5 (Drosophila)
NM_003069	SWI; ISWI; SWI2; SNF2L; SNF2L1; SNF2LB; NURF140; FLJ41547; DKFZp686D1623	SWI/SNF related, matrix associated, actin dependent regulator of chromatin, subfamily a, member 1
AA749101	IFITM1	Interferon induced transmembrane protein 1 (9-27)
BE962749		601656143R1 NIH_MGC_66 Homo sapiens cDNA clone IMAGE:3855754 3', mRNA sequence.
AI676241		CDNA clone IMAGE:5262734
AL136139	dJ761I2.1	match: proteins: Tr:Q61140 Tr:Q63766 Tr:O35177 Tr:Q14511 Tr:Q9YHC0; Human DNA sequence from clone RP4-761I2 on chromosome 6 Contains 3' part of the gene for enhancer of filamentation (HEF1), ESTs, STSs and CpG islands, complete sequence.
NM_001902	MGC9471	cystathionase (cystathionine gamma-lyase)
NM_002937	RNS4; MGC9306	ribonuclease, RNase A family, 4
AW134535	CCNG2	Cyclin G2
N21643	FBXO32	CDNA FLJ39585 fis, clone SKMUS2006633
NM_004772	P311; PTZ17; D4S114; PRO1873	chromosome 5 open reading frame 13
BE972723	C4orf34	Chromosome 4 open reading frame 34
BE966604		601660289R1 NIH_MGC_71 Homo sapiens cDNA clone IMAGE:3905950 3', mRNA sequence.
NM_019093	UGT1C	UDP glucuronosyltransferase 1 family, polypeptide A3
NM_000204	FI; IF; KAF; C3b-INA; factor I	complement factor I
NM_002534	OIAS; IFI-4; OIASI	2',5'-oligoadenylate synthetase 1, 40/46kDa
D50918	SEP2; SEPT2; KIAA0128; MGC16619; MGC20339; RP5-876A24.2	septin 6
AW612403	C14orf45	Transcribed locus
AW474434	TNFSF10	Tumor necrosis factor (ligand) superfamily, member 10
U73191	IRKK; KIR1.3; KIR4.2; MGC13584	potassium inwardly-rectifying channel, subfamily J, member 15
AF060922	NIX; BNIP3a	BCL2/adenovirus E1B 19kDa interacting protein 3-like
NM_015271	RNF86; KIAA0517	tripartite motif-containing 2
NM_003069	SWI; ISWI; SWI2; SNF2L; SNF2L1; SNF2LB; NURF140; FLJ41547; DKFZp686D1623	SWI/SNF related, matrix associated, actin dependent regulator of chromatin, subfamily a, member 1
AI806592	SLA/LP	MRNA; cDNA DKFZp434B1417 (from clone DKFZp434B1417)
AL354872		
BC001356	IFP35; FLJ21753	interferon-induced protein 35
AW086021	ZNF323	Zinc finger protein 323
AW902062		Transcribed locus
AK026426	SWI; ISWI; SWI2; SNF2L; SNF2L1; SNF2LB; NURF140; FLJ41547; DKFZp686D1623	SWI/SNF related, matrix associated, actin dependent regulator of chromatin, subfamily a, member 1
W57613		Transcribed locus
BC009800	SEC24B	CDNA clone IMAGE:4049873
BF125756	GABARAPL1	GABA(A) receptor-associated protein like 1
AV700050		AV700050 GKC Homo sapiens cDNA clone GKCECA03 3', mRNA sequence.
U57059	TL2; APO2L; CD253; TRAIL; Apo-2L	tumor necrosis factor (ligand) superfamily, member 10
AF118886	FLJ40431	vav 3 oncogene
NM_006994	BTF3	butyrophilin, subfamily 3, member A3
NM_003810	TL2; APO2L; CD253; TRAIL; Apo-2L	tumor necrosis factor (ligand) superfamily, member 10
AL136693	DCYTB; FRRS3; FLJ23462	cytochrome b reductase 1
NM_014398	LAMP; CD208; DCLAMP; TSC403; DC-LAMP	lysosomal-associated membrane protein 3
BC043357	DLGAP1	Homo sapiens, clone IMAGE:3883659, mRNA
AF019214	FLJ16340	HMG-box transcription factor 1
AI738802	CDK11	Transcribed locus
AI760166	ALPK1	Alpha-kinase 1
NM_013943	H1; huH1; p64H1; CLIC4L; FLJ38640; DKFZP566G223	chloride intracellular channel 4
AL136771	Men; ELL1; C19orf17; DKFZp434I1916	elongation factor RNA polymerase II
AF087847	ATG8; GEC1; APG8L	GABA(A) receptor-associated protein like 1
AA584310	CTHRC1	Collagen triple helix repeat containing 1
AI761728	RNASE4	Ribonuclease, RNase A family, 4
NM_006472	THIF; VDUP1; HHCPA78; EST01027	thioredoxin interacting protein
BG502305	DZIP3	Zinc finger DAZ interacting protein 3
M18767	C1S	complement component 1, s subcomponent
BF038548	PAM	Peptidylglycine alpha-amidating monooxygenase
AI927964	NUDT7	Nudix (nucleoside diphosphate linked moiety X)-type motif 7
AW235612	ATXN1	Ataxin 1
BE550967		Transcribed locus
AW014593	GBP1	Guanylate binding protein 1, interferon-inducible, 67kDa
BC002666	GBP1	guanylate binding protein 1, interferon-inducible, 67kDa
NM_003304	TRP1; HTRP-1; MGC133334; MGC133335	transient receptor potential cation channel, subfamily C, member 1
NM_017577	FLJ35862; FLJ40464; DKFZp434C0328; DKFZp686K06117	GRAM domain containing 1C
AJ001696	HUR7; PI13; headpin; MGC126870	serpin peptidase inhibitor, clade B (ovalbumin), member 13
AF109196	H1; huH1; p64H1; CLIC4L; FLJ38640; DKFZP566G223	chloride intracellular channel 4
BC037317	KIAA1107	KIAA1107
AL137517	MGC119595; MGC119596; MGC119597	SLIT and NTRK-like family, member 6
AA745978		Full length insert cDNA clone YQ54B06
AK025872	CAGH39	trinucleotide repeat containing 8
AI439556	TXNIP	Thioredoxin interacting protein
NM_002774	hK6; Bssp; Klk7; SP59; ZYME; PRSS9; PRSS18; MGC9355; NEUROSIN	kallikrein-related peptidase 6
NM_016621	BHC80; BM-006; KIAA1696	PHD finger protein 21A
AF279899	B4-2; PRR2; PROL2; PNAS-145; RP11-63L7.5	proline-rich nuclear receptor coactivator 1
NM_018328	FLJ11113; FLJ30517; KIAA1461	methyl-CpG binding domain protein 5
NM_003022	SH3BGR; MGC117402	SH3 domain binding glutamic acid-rich protein like
NM_000585	IL-15; MGC9721	interleukin 15
AA142966	PJA2	Praja 2, RING-H2 motif containing
N32035	STX7	Syntaxin 7
U64317	CASL; HEF1; CAS-L; dJ49G10.2; dJ761I2.1	neural precursor cell expressed, developmentally down-regulated 9
M95929	PHOX1	Human homeobox protein (PHOX1) mRNA, 3' end.
BC000836	CGI-127	yippee-like 5 (Drosophila)
NM_006113	FLJ40431	vav 3 oncogene
BF435952	C14orf28	Chromosome 14 open reading frame 28
AF237813	GABAT; NPD009; GABA-AT	4-aminobutyrate aminotransferase
NM_013314	BASH; Ly57; SLP65; BLNK-s; SLP-65; MGC111051	B-cell linker
AI638279	BRWD1	Bromodomain and WD repeat domain containing 1
AI763378	EHF	Ets homologous factor
AU156421		CDNA FLJ13457 fis, clone PLACE1003343
BC016050	BITE; FLJ13036	centrosomal protein 70kDa
AI913749	PLEKHH2	Pleckstrin homology domain containing, family H (with MyTH4 domain) member 2
AW779950	SLC25A27	Solute carrier family 25, member 27
AA778688	C7orf31	Chromosome 7 open reading frame 31
NM_000240	MAOA	monoamine oxidase A
AF124438	ESE3; ESEJ	ets homologous factor
NM_000463	GNT1; UGT1; UDPGT; UGT1A; HUG-BR1	UDP glucuronosyltransferase 1 family, polypeptide A1
AB028951	CDK11; KIAA1028; bA346C16.3	cell division cycle 2-like 6 (CDK8-like)
BE221212		hu22a05.x1 NCI_CGAP_Mel15 Homo sapiens cDNA clone IMAGE:3170768 3' similar to gb:K01228 PROCOLLAGEN ALPHA 1(I) CHAIN PRECURSOR (HUMAN);, mRNA sequence.
AF077048	HSPC116; DKFZp686F03273	single-stranded DNA binding protein 2
AW297656	CDRT4	CMT1A duplicated region transcript 4
NM_006393	LNEBL; bA56H7.1; MGC119746; MGC119747	nebulette
AA732944		zg78d04.s1 Soares_fetal_heart_NbHH19W Homo sapiens cDNA clone IMAGE:399463 3', mRNA sequence.
AI375915	PSMB9	Proteasome (prosome, macropain) subunit, beta type, 9 (large multifunctional peptidase 2)
BF510429	CLINT1	Clathrin interactor 1
NM_000950	PRGP1	proline rich Gla (G-carboxyglutamic acid) 1
NM_005460	SYPH1; MGC39814	synuclein, alpha interacting protein (synphilin)
NM_005589	MMSDH; MMSADHA; MGC40271	aldehyde dehydrogenase 6 family, member A1
BF346014	IDS	Iduronate 2-sulfatase (Hunter syndrome)
NM_000197	EDH17B3	hydroxysteroid (17-beta) dehydrogenase 3
U25804	TX; ICH-2; Mih1/TX; ICEREL-II; ICE(rel)II	caspase 4, apoptosis-related cysteine peptidase
N71923		yz36g02.s1 Morton Fetal Cochlea Homo sapiens cDNA clone IMAGE:285170 3', mRNA sequence.
NM_019903	ADDL	adducin 3 (gamma)
U90552	BTF5; BT3.1; CD277; MGC141880	butyrophilin, subfamily 3, member A1
NM_022168	Hlcd; MDA5; MDA-5; IDDM19; MGC133047	interferon induced with helicase C domain 1
NM_014349	CG12-1; APOLIII	apolipoprotein L, 3
AI763123	ADD3	Adducin 3 (gamma)
AI971089	CRSP2	Cofactor required for Sp1 transcriptional activation, subunit 2, 150kDa
AA577672	DTX3L	Deltex 3-like (Drosophila)
AW043602	KIAA1946	KIAA1946
AJ001698	HUR7; PI13; headpin; MGC126870	serpin peptidase inhibitor, clade B (ovalbumin), member 13
BE545756	ADD3	Adducin 3 (gamma)
AB037797	TLIMP; KIAA1376	arrestin domain containing 3
NM_006055	p40; GPR69A	LanC lantibiotic synthetase component C-like 1 (bacterial)
N92498	PDCD4	Programmed cell death 4 (neoplastic transformation inhibitor)
NM_004223	RIG-B; UBCH8; MGC40331	ubiquitin-conjugating enzyme E2L 6
AA056548	PARP14	Poly (ADP-ribose) polymerase family, member 14
NM_152701	FLJ16398; FLJ33876; FLJ33951; DKFZp313D2411	ATP-binding cassette, sub-family A (ABC1), member 13
AI419423	PAPOLG	Poly(A) polymerase gamma
NM_005981	SAS	tetraspanin 31
U13700	ICE; P45; IL1BC	caspase 1, apoptosis-related cysteine peptidase (interleukin 1, beta, convertase)
BC000758	C6orf80; CCRL1AP; MGC131913; DKFZp586D0623	coiled-coil domain containing 28A
M55580	SAT; DC21; KFSD; SSAT; SSAT-1	spermidine/spermine N1-acetyltransferase 1
AK025862	ECGP; GP96; TRA1; GRP94	heat shock protein 90kDa beta (Grp94), member 1
BF126274	STX17	Full-length cDNA clone CS0DB009YL20 of Neuroblastoma Cot 10-normalized of Homo sapiens (human)
AF129536	FBG2; FBS2; FBX6; Fbx6b	F-box protein 6
NM_018373	Arip2; OMP25; FLJ11271	synaptojanin 2 binding protein
AA628398	STARD4	START domain containing 4, sterol regulated
AU151788	C10orf75	Chromosome 10 open reading frame 75
K01228	OI4	collagen, type I, alpha 1
AV734793	ZDBF2	Zinc finger, DBF-type containing 2
AI478747	FAM102B	Family with sequence similarity 102, member B
NM_014171	HSPC139	cysteine-rich PDZ-binding protein
NM_020987	FLJ45464; ANKYRIN-G	ankyrin 3, node of Ranvier (ankyrin G)
AI638420	CLIC4	Chloride intracellular channel 4
NM_144506	NP; HNP; NRPN; PRSS19; TADG14	kallikrein-related peptidase 8
AF201932	FBS; DC10; FBX8	F-box protein 8
AW973253		Transcribed locus
NM_003265	CD283	toll-like receptor 3
BG434174	ALF	TFIIA-alpha/beta-like factor
AF194973	DINP; POLQ; DINB1	polymerase (DNA directed) kappa
NM_000919	PAL; PHM	peptidylglycine alpha-amidating monooxygenase
NM_014845	SAC3; hSac3; dJ249I4.1; RP1-249I4.1	KIAA0274
AI963713	ENAH	Enabled homolog (Drosophila)
AL157398		
BC002832	BTF4; BT3.2; BT3.3	butyrophilin, subfamily 3, member A2
BF685808	SESN3	Sestrin 3
BC012846	IDH; IDP; PICD	isocitrate dehydrogenase 1 (NADP+), soluble
BF111821	WSB1	WD repeat and SOCS box-containing 1
AF044221	HCG-1; PNSC1; FLJ32018; DKFZP434K151	ubiquitin-like 3
BC004130	NDP52; MGC17318	calcium binding and coiled-coil domain 2
AA824363	C9orf85	Chromosome 9 open reading frame 85
AU144005	ITGAV	CDNA FLJ11397 fis, clone HEMBA1000622
BC015714	BHC80; BM-006; KIAA1696	PHD finger protein 21A
AI342246		Transcribed locus
NM_021027	LUGP4; UDPGT; HLUGP4; UGT1AI	UDP glucuronosyltransferase 1 family, polypeptide A9
AL132665	NIX; BNIP3a	BCL2/adenovirus E1B 19kDa interacting protein 3-like
NM_024498	HPF9; H-plk; MGC22613	zinc finger protein 117
AI148659	HEG1	HEG homolog 1 (zebrafish)
AW469184	MOSPD2	Motile sperm domain containing 2
NM_000050	ASS; CTLN1	argininosuccinate synthetase 1
AW304174	CTBS	Chitobiase, di-N-acetyl-
NM_019891	ERO1LB	ERO1-like beta (S. cerevisiae)
NM_030934	MST070; MSTP070; MGC25112; MGC57134; bG120K12.3	chromosome 1 open reading frame 25
NM_016816	OIAS; IFI-4; OIASI	2',5'-oligoadenylate synthetase 1, 40/46kDa
AV700415	LOC54103	Hypothetical protein LOC54103
AL573058	C1R	Complement component 1, r subcomponent
AF087481	PUT1; PLU-1; FLJ10538; FLJ12459; FLJ12491; FLJ16281; FLJ23670; RBBP2H1A	jumonji, AT rich interactive domain 1B
NM_004457	ACS3; FACL3; PRO2194	acyl-CoA synthetase long-chain family member 3
BF058944	SCAMP1	Secretory carrier membrane protein 1
NM_024744	CARF; FLJ21579; NYD-SP24; DKFZp667N246	amyotrophic lateral sclerosis 2 (juvenile) chromosome region, candidate 8
AA923354	MAOA	Monoamine oxidase A
AK092103	C6orf199	Chromosome 6 open reading frame 199
NM_003113	FLJ00340; FLJ34579; DKFZp686E07254	SP100 nuclear antigen
AL137958	RPL5	Ribosomal protein L5
NM_006307	DRS; ETX1	sushi-repeat-containing protein, X-linked
NM_173487	FLJ33703	chromosome 4 open reading frame 33
NM_016008	LIC3; D2LIC; CGI-60; DKFZP564A033	dynein, cytoplasmic 2, light intermediate chain 1
AV691323	UGT1A6	UDP glucuronosyltransferase 1 family, polypeptide A6
AL136680	FLJ10961; DKFZp686E0974; DKFZp686L15228	guanylate binding protein 3
AB041261	IPLA2G; IPLA2-2; IPLA2(GAMMA)	patatin-like phospholipase domain containing 8
AV704962	DESP4; ERG25; MGC104344	sterol-C4-methyl oxidase-like
